# Improved approach for electric vehicle rapid charging station placement and sizing using Google maps and binary lightning search algorithm

**DOI:** 10.1371/journal.pone.0189170

**Published:** 2017-12-08

**Authors:** Md. Mainul Islam, Hussain Shareef, Azah Mohamed

**Affiliations:** 1 Department of Electrical and Electronic Engineering, Uttara University, Uttara Model Town, Dhaka, Bangladesh; 2 Department of Electrical Engineering, United Arab Emirates University, 1 Al-Ain, UAE; 3 Department of Electrical, Electronic, and Systems Engineering, Universiti Kebangsaan Malaysia, Selangor, Malaysia; Chongqing University, CHINA

## Abstract

The electric vehicle (EV) is considered a premium solution to global warming and various types of pollution. Nonetheless, a key concern is the recharging of EV batteries. Therefore, this study proposes a novel approach that considers the costs of transportation loss, buildup, and substation energy loss and that incorporates harmonic power loss into optimal rapid charging station (RCS) planning. A novel optimization technique, called binary lightning search algorithm (BLSA), is proposed to solve the optimization problem. BLSA is also applied to a conventional RCS planning method. A comprehensive analysis is conducted to assess the performance of the two RCS planning methods by using the IEEE 34-bus test system as the power grid. The comparative studies show that the proposed BLSA is better than other optimization techniques. The daily total cost in RCS planning of the proposed method, including harmonic power loss, decreases by 10% compared with that of the conventional method.

## Introduction

Electric vehicles (EVs) have gained increasing attention in recent years because of the enormous amount of carbon dioxide gas released from conventional vehicles. Dependency on imported crude oil and the depletion of fossil fuel resources lead to the search for alternative energy sources that are efficient, economical, and environment friendly for transportation systems. EVs do not generate any form of emission and pollution. However, the main obstacle to the widespread deployment of EVs from the customer perspective is the lack of charging stations (CSs). EV users urgently need a charging solution for long-distance driving. Therefore, a sufficient number of rapid CSs (RCSs) are important for rapid charging and for alleviating charge anxiety. Rapid charging may cause massive loading in a distribution network when EVs connect to an RCS. Therefore, finding an optimal location and sizing for RCSs that generate few adverse impacts on power distribution grids and that reduce transportation energy loss (TEL) is an essential requirement for the widespread deployment of EVs.

Recent studies have focused on the optimal placement and sizing of public CSs. These studies have considered two aspects: economics and power grid-related concepts. From the economic perspective, the authors of [1] developed a CS placement method based on grid partition that minimizes transportation cost by using a genetic algorithm (GA) to access a CS. Nonetheless, cost functions, such as fixed, land, and operating costs, were disregarded in optimizing the system. In [2], particle swarm optimization (PSO) was applied to identify an optimal location for a CS based on construction and running costs and by considering geographic information and traffic flow as constraint conditions. A mixed-integer programming model was proposed in [3] to determine CS locations for diminishing range anxiety and minimizing traveling distance from an EV to a CS. The authors of [4] developed a model for CS placement and sizing that relies on fast charging demand distribution and road network structure; they applied CPLEX software to solve the problem.

In [5], a multi-objective, multivariate optimal planning model was proposed in terms of investment costs and feeder energy losses with other constraint conditions to solve the optimal CS planning problem that includes power system issues. The proposed approach was tested on the IEEE 33-node distribution system by using an improved GA and then compared with a traditional GA. A GA with the Pareto optimal front was utilized in [6] to minimize traveling and power loss costs using the IEEE 14-bus test system. A GA was also adopted in [7] to find the best parameters among three different models of ultra-capacitors for an EV energy management system based on model accuracy, complexity, and robustness [7]. Meanwhile, the state of charge (SOC) of ultra-capacitors was studied in [8]. A cost model was suggested in [9] by considering the operating costs of a CS, the investment costs of a distribution transformer, and network loss. This model considers different constraints, such as the distance between a substation and EV location, the number of EVs, and the installation costs of CS. PSO was applied to optimize the system. Nevertheless, the prediction of charging demand was neglected in optimizing the model. The authors of [10] proposed a cost-optimal control model to minimize the daily operational expenses of a plug-in hybrid EV charging from a grid, fuel consumption during on-road driving, and battery aging by using convex programming (CP). Similar studies were conducted in [11]; however, integrated CP was utilized to improve computational efficiency. A traffic-constrained poly-objective pattern was introduced in [12] by considering traffic system and power loss for optimal CS placement. This method applied data employment analysis to determine the best candidate solution and cross-entropy algorithm for solving the optimization problem. These techniques effectively reduce power loss, voltage deviation, and travel distance to a CS. The authors of [13] developed an EV model that combined constant power and voltage-dependent load to find optimal CS locations in a power grid based on voltage stability margins, grid power loss, and cable flow ratings. Similarly, an EV model was developed in MATLAB Simulink in [14] with a combination of constant power and voltage-dependent and negative exponential components. The model was simulated on the IEEE 43-bus test distribution system. In [15], the authors found optimal CS sites by considering environmental factors and maximum service coverage. A cost function, including power system loss cost, was developed to determine the optimal sizing of CSs. The method was utilized on the IEEE 123-node test feeder system by adopting a modified primal–dual interior point algorithm. The authors of [16] identified an optimal CS location on a distribution grid by minimizing total costs and real power loss while adopting power system security and traffic flow as constraints. Ant colony optimization was used to find the best CS location on an existing distribution grid. The IEEE 69-bus system was utilized to validate this technique. Existing optimal placement and sizing approaches for CSs are discussed in [17, 18]; and different types of impacts, current issues, challenges of EVs, and technical development of batteries are described in detail in [18, 19]. Besides, the utilization of plug-in EVs along with home battery storage system for smart home energy management is presented in [20, 21].

Previous authors have mainly focused on economic parameters and neglected other parameters, such as a real city transportation network for calculating time and distance from an EV to a CS and SOC for calculating transportation and harmonic power losses caused by EV charging [22]. Accordingly, a novel approach that considers transportation loss (which introduces Google Maps JavaScript API to calculate the real time and distance from the current location of an EV to a CS in consideration of SOC), buildup (BU) cost (which incorporates the costs of the distribution transformer, underground cable, and charger in addition to other costs), and grid power loss cost (which comprises harmonic power loss) is proposed in this study to determine the optimal placement and sizing of RCSs. A new optimization technique, called binary lightning search algorithm (BLSA) [23], is utilized as an optimization tool to solve the RCS planning problem. A comparative study is conducted using the method presented in [24], and an assessment was performed to highlight the importance of factors that affect the RSC planning considered in the proposed method.

## Problem formulation for optimal RCS planning

Decision vector, objective functions, and optimization constraints are important elements for binary optimization. These elements, with respect to the RCS placement and sizing problem, are described in the following subsections.

### Decision vector

Each possible RCS position in a road network system during the optimization process is selected by using the decision vector, called the RCS placement (*RCSP*) vector. *RCSP* is an n bit binary string, in which bit 0 (zero) indicates that no RCS needs to be constructed and bit 1 (one) indicates that an RCS should be installed at a specified site in the road network. Hence, the *RCSP* vector can be mathematically represented as

$$RCSP\;(i)=\left\{ \begin{array}{ll} \;1, & \mathrm{if}\;\mathrm{RCS}\;\mathrm{is}\;\mathrm{required}\\ \;0, & \mathrm{otherwise} \end{array}\right.\;i=1,2,3,\ldots ,\;N_{RCS},$$ (1)

where *N*_*RCS*_ is the number of RCSs that are considered possible RCS sites.

### Multi-objective functions

For the optimal placement and sizing of RCS, different cost functions, namely, TEL, BU, and substation energy loss (SEL) costs, are considered.

#### TEL cost

**Fig 1:**
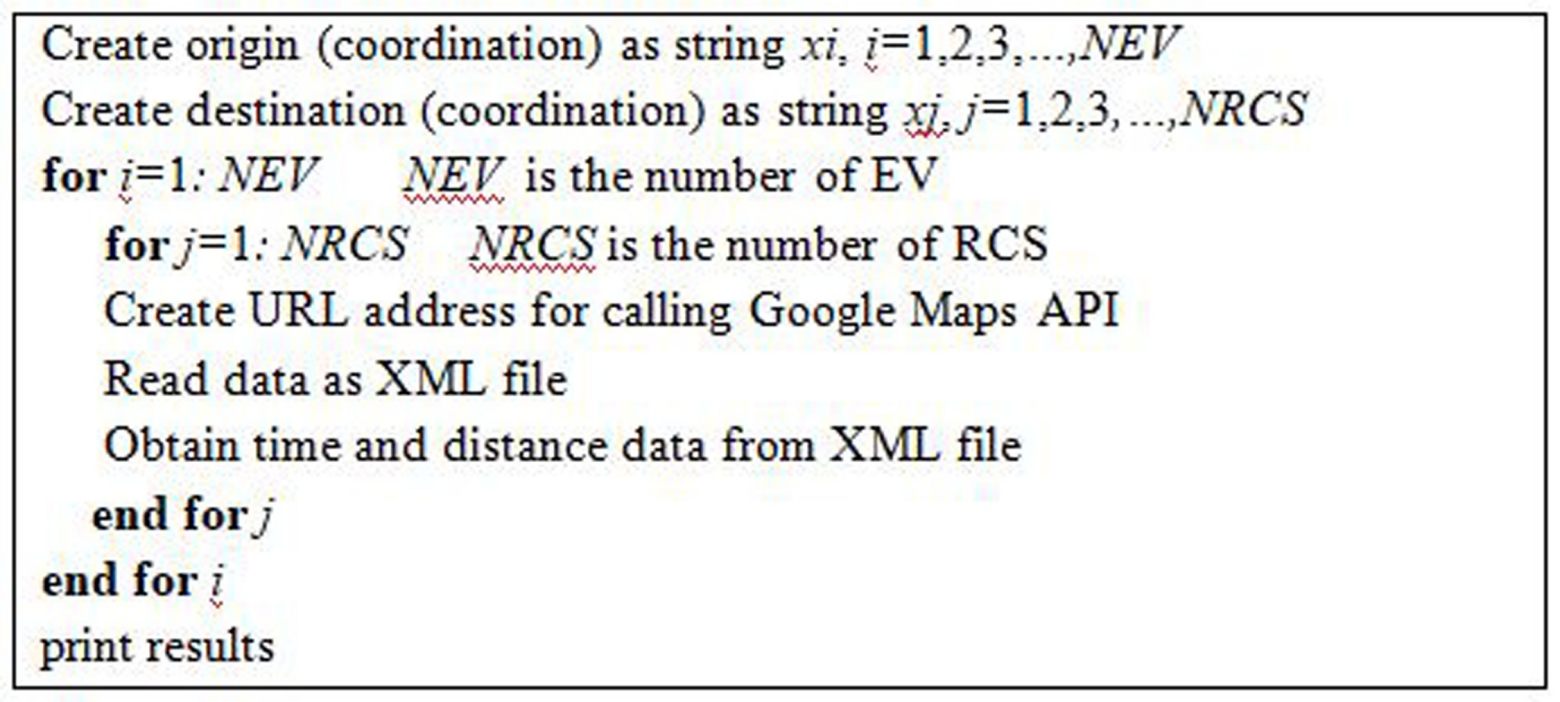
Time and distance calculation. Pseudo code for attaining time and distance from an EV to RCSs using Google Maps API.

EV users have to travel a long distance by utilizing an enormous amount of energy to reach an RCS, which can be considered TEL. Google Maps JavaScript API is used in this study to obtain realistic data, such as time and distance from the origin, i.e., the current location of an EV, to the destination, i.e., an RCS. Fig 1 shows the pseudo code of the Google Maps API system for calculating distance and time. In the figure, the URL address holds links that have the information of the distance matrix, i.e., time and distance. Table 1 presents sample time and distance data from an EV to various RCS destinations achieved using Google Maps API. Reaching an RCS located at a long distance within a short time is occasionally possible, as shown in Table 1. Hence, time is considered in TEL cost calculation due to environmental factors, such as traffic on the road.

**Table 1:** Sample EV-to-RCS data obtained using Google Maps API.

EV Origin	RCS Destinations	Time (h)	Distance (km)
‘3.000,101.7450’	‘3.048,101.7060	0.350	13.70
	‘2.951,101.7882’	0.367	13.10
	‘3.061,101.7751’	0.317	13.70

Hence, time taken (*t*_*i*_) from a specific *EV*_*i*_ location to all available RCSs can be represented as

$$T_{i}=[\begin{array}{ccccc} t_{1} & t_{2} & t_{3} & \ldots & t_{N_{RCS}} \end{array}],$$ (2)

and the minimum time (*T*_*i_min*_) from the *EV*_*i*_ location to the nearest RCS can be obtained as

$$T_{i\_min}=min\;(T_{i}),$$ (3)

Obtaining *T*_*i_min*_ and the current SOC of *EV*_*i*_ ensures that the remaining charge percentage in the *EV*_*i*_ battery in terms of SOC at an RCS can be expressed as

$$SOC_{ts}^{i}=SOC_{cp}^{i}-D_{bat}\times \frac{T_{i\_min}}{C_{bat}},$$ (4)

where $SOC_{ts}^{i}$ is the SOC after time *T*_*i_min*_, $SOC_{cp}^{i}$ is the SOC at the current position, *D*_*bat*_ is the battery discharge rate, and *C*_*bat*_ is the battery capacity.

In this study, an EV is assumed to stop when the minimum SOC (*SOC*_*min*_) limit is reached. This limit is predefined as 20%. Therefore, an approximate number of EVs that utilize a specific RCS ($N_{EV}^{RCS_{j}}$) based on the calculated SOC ($SOC_{ts}^{i}$) after time *T*_*i_min*,_ and *SOC*_*min*_ for all EVs can be obtained as

$$N_{EV}^{RCS_{j}}=\sum_{i=1}^{N_{EV}}\mathrm{RCSP}(1+sign\;(SOC_{ts}^{i}-SOC_{min}))\;i=1,2,3,\ldots ,N_{EV},$$ (5)

where ***RCSP*** is the RCS placement decision vector, $SOC_{ts}^{i}$ is the SOC after time *T*_*i_min*_, and *N*_*EV*_ is the number of EVs.

If $\left(SOC_{ts}^{i}-SOC_{min}\right)$ is negative, then *EV*_*i*_ cannot reach the RCS. Hence, a high cost should be included for other services (e.g., tow trucks) to tow the EV to the RCS. The *TEL* cost for *EV*_*i*_ to access the nearest RCS can be expressed as

$$TEL_{i}=P_{E}\times D_{bat}\times T_{i\_min},$$ (6)

The *TEL* for *EV*_*i*_ that cannot access the nearest RCS when SOC reaches below *SOC*_*min*_ can be represented as

$$TEL_{i}=l_{i}\times C_{EV},$$ (7)

where *P*_*E*_ is the electricity price in $/ kWh, *D*_*bat*_ is the battery discharge rate in kWh, *L*_*i*_ is the distance from *EV*_*i*_ to the nearest RCS in km, and *C*_*EV*_ is the extra cost in $/km.

The normalized total *TEL* cost (*TEL*_*norm*_) is expressed as

$$TEL_{norm}=\frac{\sum_{i=1}^{N_{EV}}TEL_{i}}{TEL^{max}}\;i=1,2,3,\ldots ,N_{EV},$$ (8)

where *TEL*^*max*^ is the maximum *TEL* cost when a single RCS is selected as the optimal RCS site.

#### BU cost

**Fig 2:**
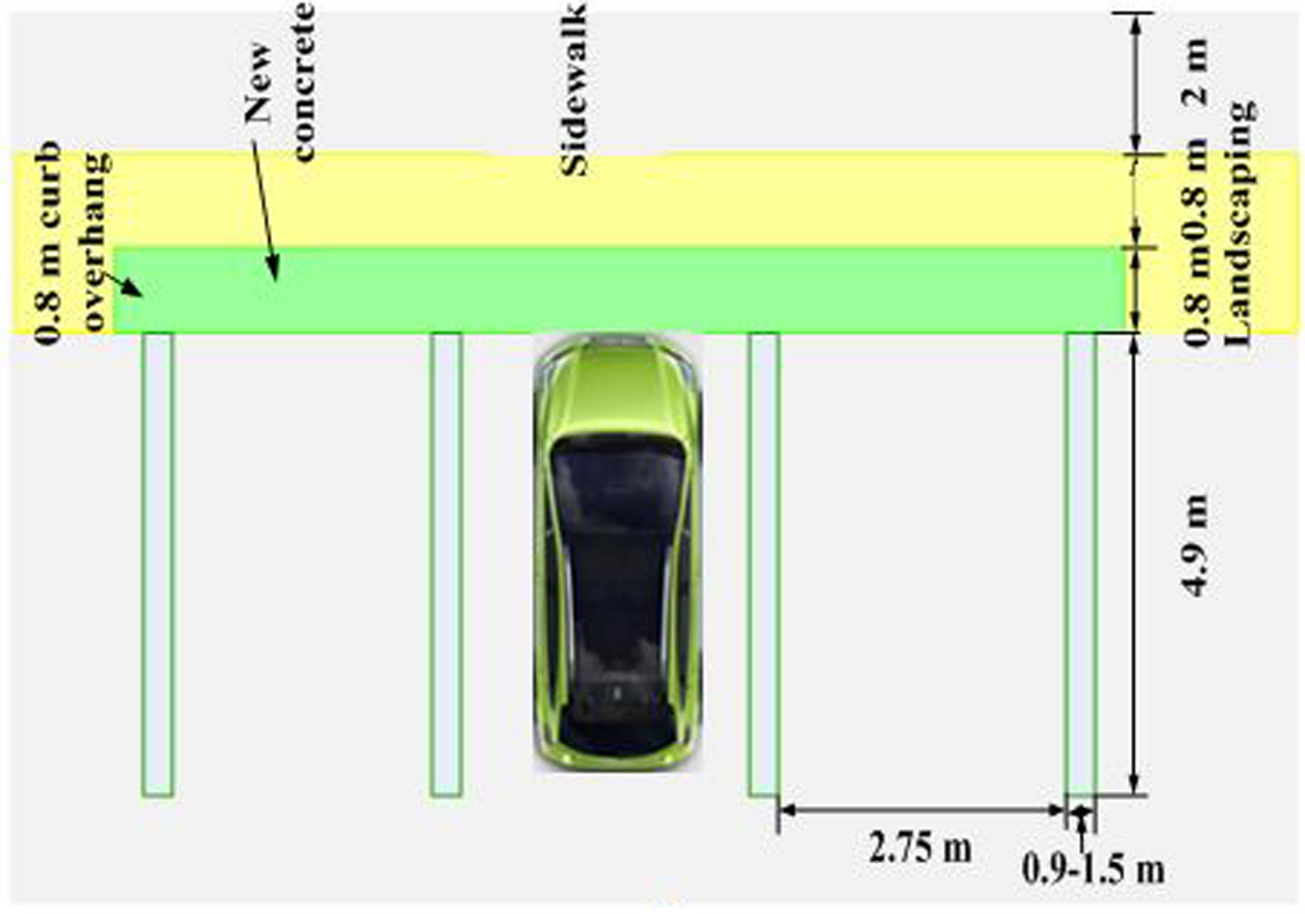
CS layout.

Station BU cost incorporates the costs of land, chargers, and distribution transformers. This cost should also include operational and underground distribution cable costs. As shown in Fig 2, for every vehicle to access a charger, a station generally needs a 4.9 m × 2.75 m area and will overhang the curb by up to 0.08 m. Parking vehicles should avert the EV supply equipment, and users should cautiously move in front of vehicles. An electrical conduit to an RCS can be placed beneath the landscaping. If more than one charger is required, then a space equal to 0.9 m to 1.5 m is necessary among the chargers. In this study, a single charger area, including landscaping and sidewalk, is considered 30 m^2^, and all the investment costs of the station are assumed to be covered for 10 years. However, the total land area and equipment cost necessary for a specific RCS depend on the number of chargers to be installed. Hence, the number of chargers (*N*_*cha*_) required for *RCS*_*j*_ is obtained as

$$N_{cha}^{j}=\frac{N_{EV}^{RCS_{j}}\times N_{cha}^{max}}{N_{EV}}\;j=1,2,3,\ldots ,N_{RCS},$$ (9)

and the capacity (*S*_*cap*_) of *RCS*_*j*_ can be calculated as

$$S_{cap}^{j}=P_{cha}\times N_{cha}^{j}\;j=1,2,3,\ldots ,N_{RCS},$$ (10)

where $N_{cha}^{max}$ is the maximum permissible number of chargers for any RCS, and *P*_*cha*_ is the rated power of a charger in kW.

When the aforementioned information is considered, the *BU* cost for *RCS*_*j*_ is calculated as

$$BU_{j}=(C_{fixed}+30\times C_{lan}\times N_{cha}^{j}+S_{cap}^{j}\times C_{cha}+C_{ugc}+C_{x-former}+C_{oper}),$$ (11)

where

$$C_{ugc}=C_{con}^{j}\times D_{j},$$ (12)

$$C_{x-former}=C_{tc}^{j}\times VA_{j},$$ (13)

$$C_{oper}=C_{mms}^{j}\times RCS_{j},$$ (14)

where station fixed cost *C*_*fixed*_ implies that the cost associated with facilities should establish *RCS*_*j*_, *C*_*lan*_ is the land cost based on local price and land shape in $/m^2^, *C*_*cha*_ is the charger BU cost in $/kW, *C*_*ugc*_ denotes the distribution cable cost, and $C_{con}^{j}$ is the conductor cost of the underground cable in $/km. The conductor cost depends on the conductor diameter that relies on power drawn through it, *D*_*j*_ is the distance between *RCS*_*j*_ and the nearest substation in km, *C*_*x-former*_ is the step-down transformer cost, $C_{tc}^{j}$ is the transformer cost in $/KVA, *VA*_*j*_ is the transformer rating based on the load demand of *RCS*_*j*_, *C*_*oper*_ implies the operational cost, and $C_{mms}^{j}$ stands for the costs of materials, maintenance, and staff salaries of *RCS*_*j*_. Therefore, the normalized total *BU* cost (*BU*_*norm*_) can be derived from Eq (11) as

$$BU_{norm}=\frac{\sum_{j=1}^{N_{RCS}}BU_{j}\times R\mathrm{CSP}}{BU^{max}}\;j=1,2,3,\ldots N_{RCS},$$ (15)

where *BU*^*max*^ is the maximum cost when all RCSs are selected.

#### SEL cost

Power follows a pattern change, which is due to the nonlinear behavior of EV charging, when a new RCS is connected to a substation. An EV charging system generally injects fundamental and multiple harmonic currents into a distribution network with different magnitudes and phase angles at various frequencies. When EVs are charged, RCSs are considered loads for a substation and induce additional power loss.

The EV load (*L*_*EV*_) for *RCS*_*j*_ is calculated as

$$L_{EV}^{j}=N_{cha}^{j}\times N_{EV}^{RCS_{j}}\times P_{r},$$ (16)

where *P*_*r*_ is the power requirement for each EV battery.

Additional power loss *APL*_*EV*_ due to RCS is then obtained as

$$APL_{EV}=(TPL_{EV}+\sum_{h=3}^{N_{h}}TPL_{h})-TPL_{origin}\;h=3,5,7,\ldots ,N_{h},$$ (17)

where *TPL*_*EV*_ and *TPL*_*h*_ are the power losses with EV loads induced by fundamental and harmonic currents, respectively, when RCSs are connected to a substation with the original load; *TPL*_*origin*_ is the total power loss without connected RCS load; and *N*_*h*_ is the number of odd harmonics.

The *SEL* cost for a substation (*bus*_*j*_) with EV load is obtained as

$$SEL_{j}=APL_{EV}\times t_{ef,j}\times P_{E},$$ (18)

where *t*_*ef*, *j*_ is the effective operating hours of RCSs connected to *bus*_*j*_. *t*_*ef*_ is important to accurately calculate the power loss of a substation. Hence, *t*_*ef*_ for charging EVs via *bus*_*j*_ is formulated as

$$t_{ef,j}=(tev_{j}\times T)/tc_{j},$$ (19)

where *tev*_*j*_ indicates the total number of EVs charged via *bus*_*j*_, *tc*_*j*_ denotes the total number of chargers connected to *bus*_*j*_, and *T* is the average charging time of an EV battery.

The normalized total *SEL* cost (*SEL*_*norm*_) is expressed as

$$SEL_{norm}=\frac{\sum_{j=1}^{N_{bus}}SEL_{j}}{SEL^{max}}\;j=1,2,3,\ldots ,N_{bus},$$ (20)

where *N*_*bus*_ is the number of bus, and *SEL*^*max*^ is the maximum *SEL* cost.

The weighted sum method is used to aggregate Eqs (8), (15) and (20) to form the overall objective function. Thus, the final multi-objective function for solving the optimization problem is expressed as

$$F=w_{1}.TEL_{norm}+w_{2}.BU_{norm}+w_{3}.SEL_{norm},$$ (21)

All the sub-objective functions in Eq (21) are transformed into per-unit functions by normalizing each component to keep the values between 0 and 1. In Eq (21), *w*_*i*_ represents the fixed-weight factors defined to the individual sub-objective function, where ∑*w*_*i*_ = 1 and 0<*w*_*i*_*<*1. The same weight factors (*w*_*i*_ = 1/3) are considered for all sub-objectives to ensure equal priority.

### System constraints

The following constraints should be fulfilled during objective function evaluation to obtain the optimal number of RCSs. An RCS is essential for EVs. Therefore, at least one RCS should be selected from the predefined possible locations where RCS should be placed, as follows:

$$\sum_{j=1}^{N_{RCS}}RCS_{j}\times R\mathrm{CSP}>0\;j=1,2,3,\ldots ,N_{RCS},$$ (22)

Moreover, at least one charger should be considered for each selected RCS, as follows:

$$N_{cha}^{j}\geq RCS_{j}\times R\mathrm{CSP}\;j=1,2,3,\ldots ,N_{RCS},$$ (23)

The permitted capacity of *RCS*_*j*_ should not exceed the maximum allowable capacity for each station, as follows:

$$S_{cap}^{j}\leq S_{cap}^{jmax}\;j=1,2,3,\ldots ,N_{RCS},$$ (24)

where $S_{cap}^{jmax}$ is the maximum capacity of *RCS*_*j*_.

The allowable voltage limits for each bus to maintain power system voltage stability are regarded as

$$0.95\leq V_{j}\leq 1.05\;j=1,2,3,\ldots ,N_{bus},$$ (25)

The allowable power limits for each distribution line are regarded as

$$P_{l}\leq P_{l-\max}\;l=1,2,3,\ldots ,N_{line},$$ (26)

where *P*_*l*_ is the apparent power, line *l* must not exceed its maximum thermal limits, *P*_*l-max*_ denotes under steady-state operation, and *N*_*line*_ is the number of distribution lines.

## BLSA as an optimization tool

An important issue in an optimization algorithm is that a single algorithm cannot find the best solution for all real-world optimization problems. Hence, new optimization algorithms must be continuously searched to advance the field of computational intelligence optimization. BLSA is a novel metaheuristic optimization algorithm that was first introduced by Islam et al. [23] based on the mechanism of step leader propagation by using the concept of fast particles, also known as projectiles. In BLSA, three projectile types, namely, the transition projectiles that create the first step leader population, the space projectiles (*P*^*S*^) that attempt to become the leader, and the lead projectile (*p*^*L*^) that represents the projectile fired from the best-positioned step leader, are used. The updating procedure for space projectiles is expressed as

$$p_{i\_new}^{S}=p_{i}^{S}\pm exprand(\mu_{i}^{}),$$ (27)

where *exprand* is an exponential random number, and *μ*_*i*_ represents the distance between the lead projectile *p*^*L*^ and the space projectile $p_{i}^{S}$ under consideration.

The updated position of *p*^*L*^ at *step+1* can be written as

$$p_{i\_new}^{L}=p_{i}^{L}+normrand(\mu_{L,}^{}\sigma_{L}),$$ (28)

where *normrand* is a random number generated by the normal distribution function, and *μ*^*L*^ is the current value of *p*^*L*^ and is the scale parameter that exponentially decreases as it finds the best solution. The detailed BLSA can be found in [23]. Space and lead projectile positions are initially determined for BLSA according to Eqs (27) and (28), respectively. A suitable probability function should be defined to force the projectile to make one of two decisions, “0” or “1,” to update each projectile position in BLSA. In this study, the probability transfer function *T*_*f*_ (*p*_*i*_) is designed according to Eq (29) to map a binary search space. By contrast, the projectile position at *step+1* is updated following the probability function with a condition shown in Eq (30).

$$T_{f}(p_{i})=|\tanh(p_{i})|,$$ (29)

$$P_{i\_new}=\left\{ \begin{array}{l} \bar{p_{i}},\;\mathrm{if}\;rand\leq |T_{f}(p_{i})|\\ p_{i},\;\mathrm{otherwise} \end{array}\right.,$$ (30)

where *rand* is a uniform random variable in interval [0, 1].

**Fig 3:**
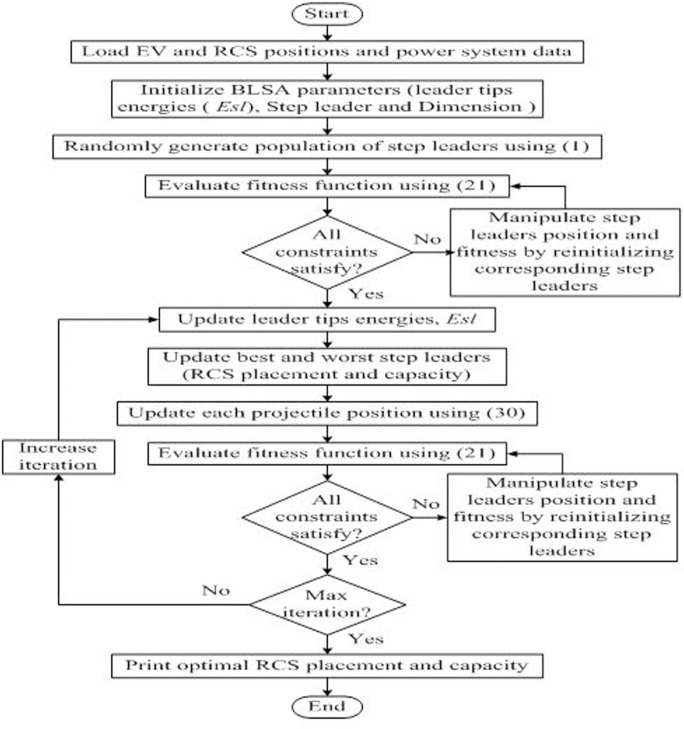
Implementation of BLSA. Implementation procedure for obtaining the optimal placement and sizing of an RCS using BLSA.

The proposed BLSA technique is utilized to minimize the proposed objective function given in Eq (21) subject to the constraints in Eqs (22)–(26) to determine the optimal placement and sizing of an RCS in the adjoining township of Bangi, Malaysia. Fig 3 presents the implementation procedure for determining the optimal placement and sizing of an RCS using BLSA.

## Description of road and power system networks

Road and power system networks are described in detail in this section. A road network is where an RCS is likely to be installed, whereas a power system network is where an RCS can obtain electric power.

### Road network

**Fig 4:**
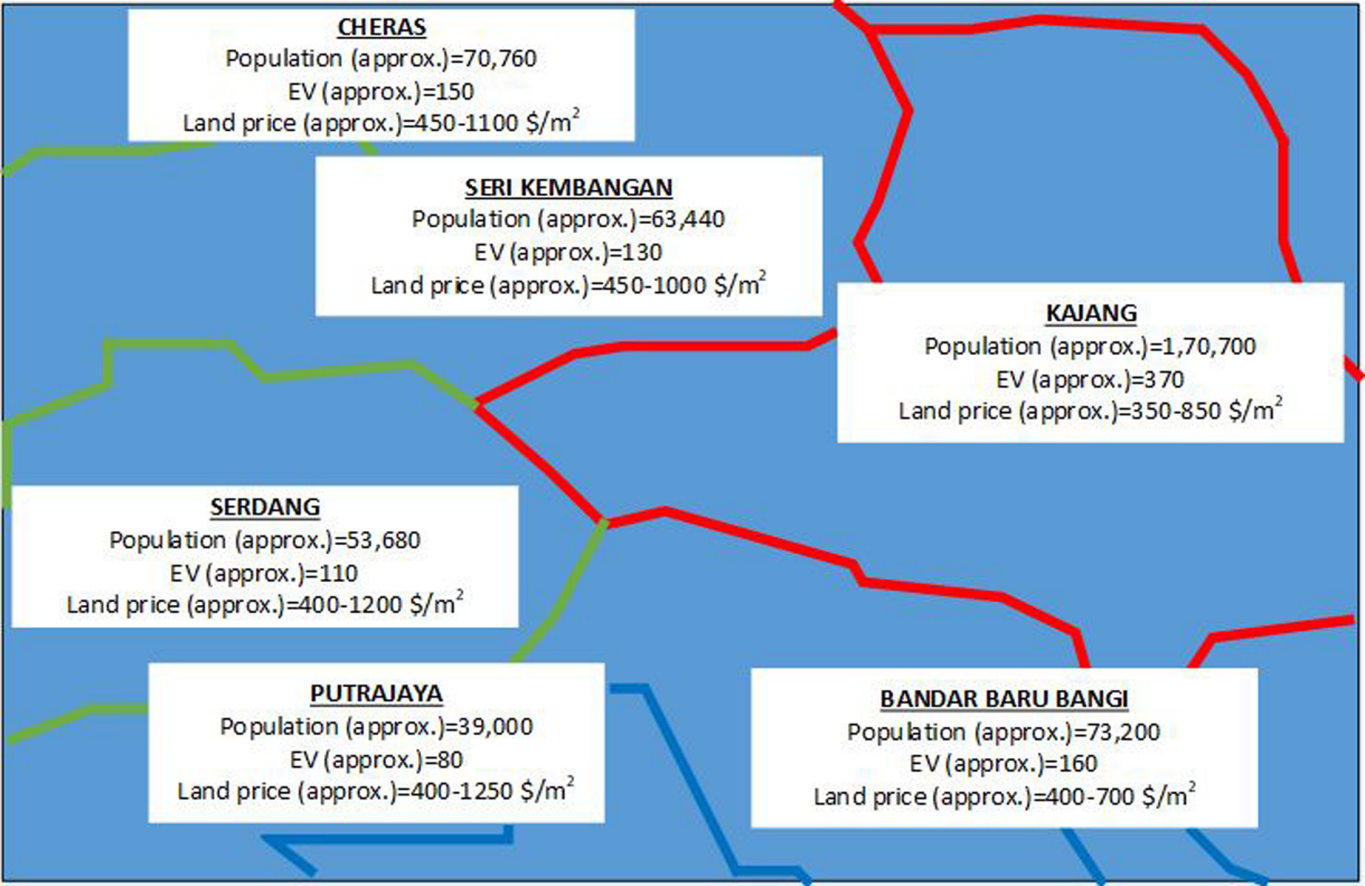
Geographical view. **View of** adjoining township of Bangi, Malaysia.

**Fig 5:**
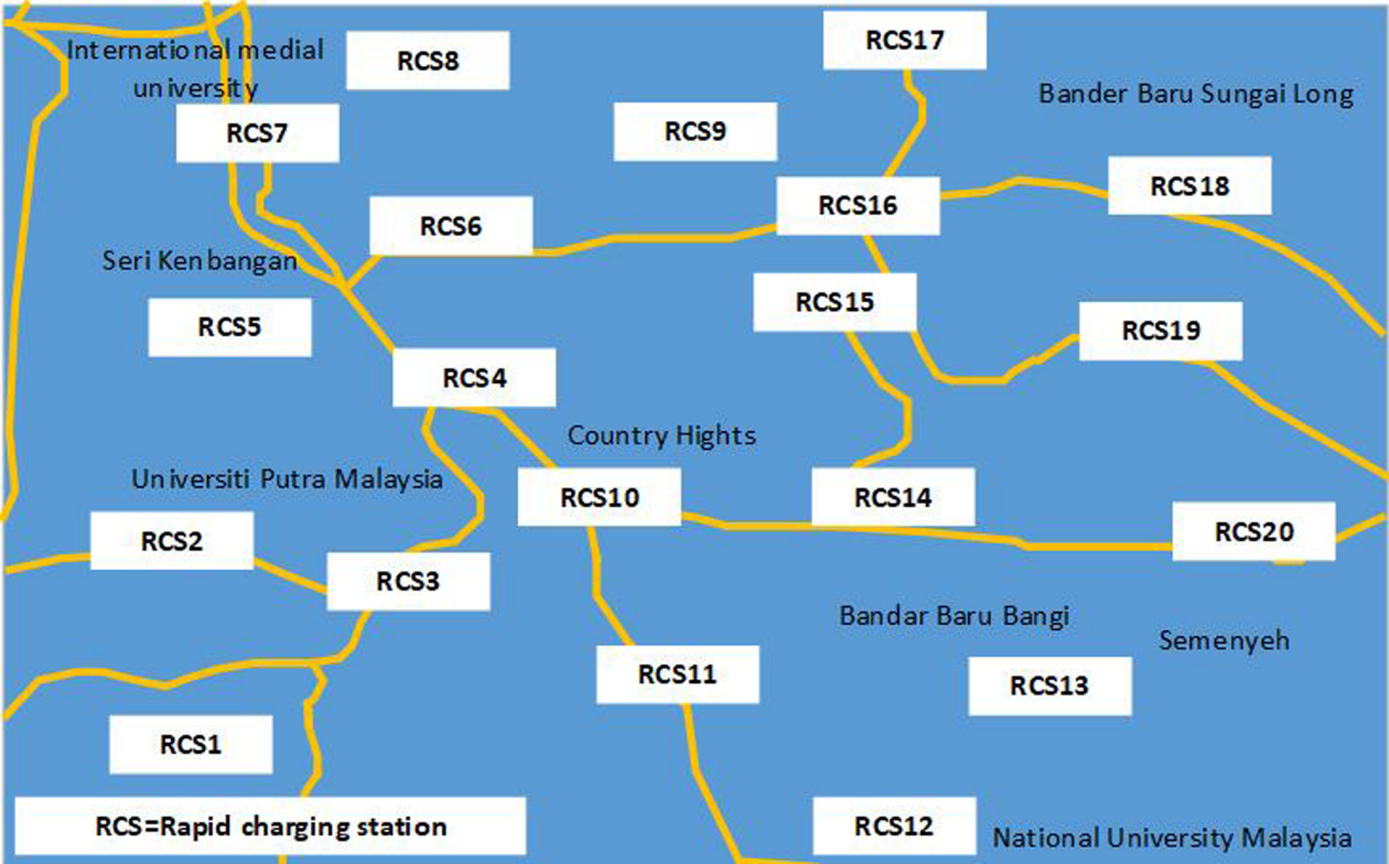
Road network. Predefined RCS locations in the adjoining township of Bangi, Malaysia.

The geographical area considered for RCS placement is the 256 km^2^ area in the adjoining township of Bangi, Malaysia, as shown in Fig 4. The figure presents the population density, number of EVs, and land price. A total of 1000 EVs (Table A in S1 File) are considered to be charged each day. EV locations in the entire area are generated depending on the population density of particular areas. The area is urban, and 20 RCSs (Table B in S1 File) are assumed to be possible if they are placed at a distance of approximately 2.5 km from one another. Fig 5 shows the road network in the adjoining township of Bangi, Malaysia considered for optimal RCS placement study. In Fig 5, the green rectangle shows the location of RCSs considered in this study. Table 2 presents the parameters utilized to calculate the objective function.

**Table 2:** Study parameters.

Parameter Name	Parameter	Value
Number of EV	*N*_*EV*_	1000
Station fixed cost	*C*_*fixed*_	12 ($/day)
Station land cost	*C*_*lan*_	0.1~0.24 ($/m^2^/day/charger)
Charger build up cost	*C*_*cha*_	0.06 ($/kW/day/charger)
Charger rated power	*P*_*cha*_	96 (kW/charger)
Conductor cost	*C*_*con*_	2.5~4 ($/km/day)
Transformer cost	*C*_*tc*_	4 ($/KVA/day)
Power factor (assumed)		0.95
Maintenance, material cost and staff salaries	*C*_*mms*_	100 ($/day)
Power requirement for each EV battery	*P*_*r*_	50 (kW)
Electricity price	*P*_*E*_	0.11 ($/kWh)
Avg. charging time	*T*	0.4 (h)
Battery capacity	*C*_*bat*_	24 (kWh)
Discharge rate	*D*_*bat*_	5.28 (kW)
Maximum permissible number of charger	$N_{cha}^{max}$	20

### Power system

**Fig 6:**
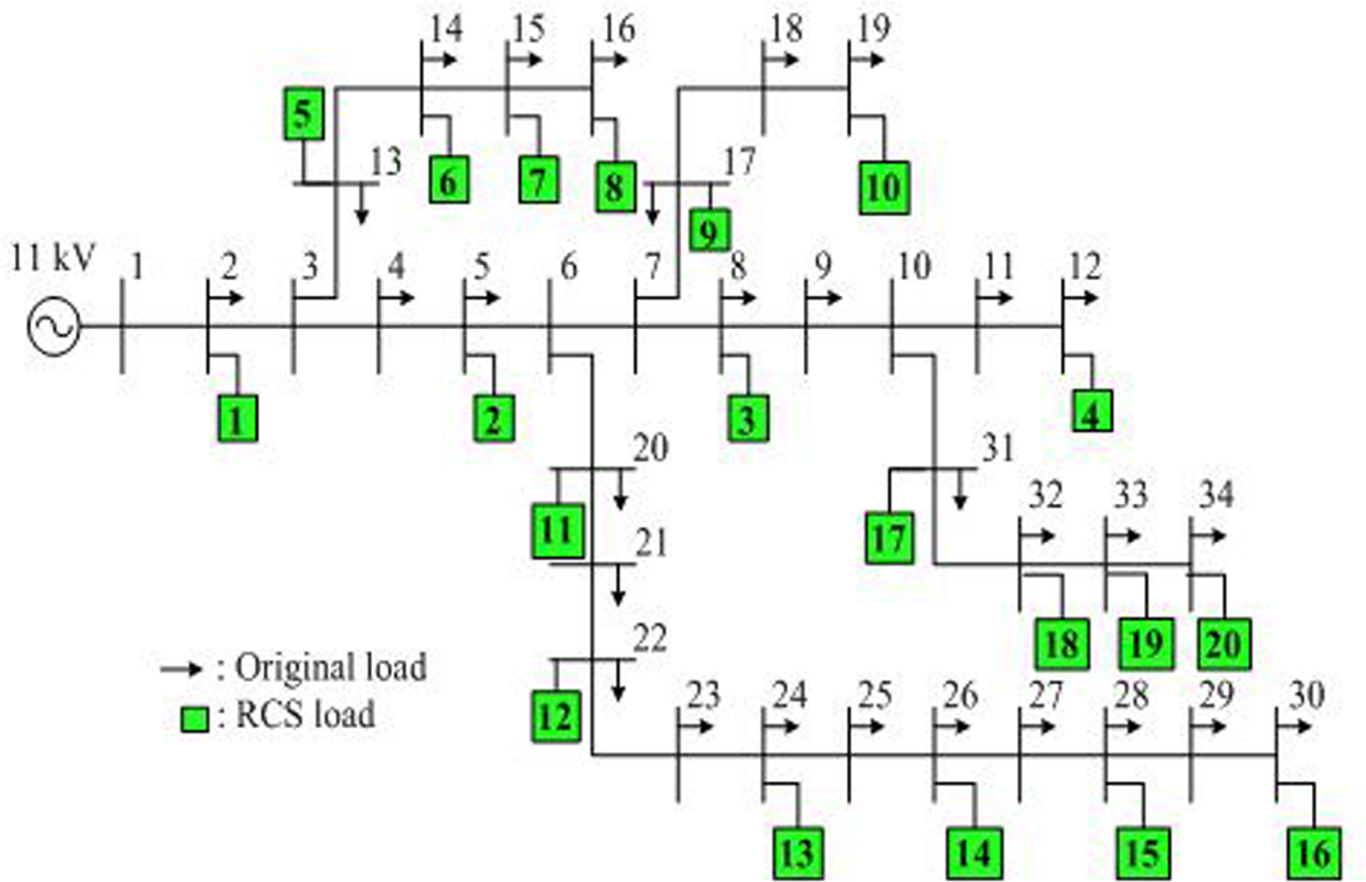
Radial bus system. IEEE 34-bus radial test system with original RCS loads.

EV charging systems produce harmonic pollution problems in the distribution system. These harmonics generally increase power losses due to the amplified root-mean-square value of the current in power lines. In this study, backward/forward sweep-based harmonic power flow is used to calculate power loss in the IEEE 34-bus test systems [25]. EVs are assumed to be connected in this bus system during rapid charging. Fig 6 shows the IEEE 34-bus radial test systems. No RCS is currently available in Malaysia; therefore, the data of injected harmonic current magnitudes and angles for the Nissan Leaf for rapid charging are obtained from Watson et al. [26].

## Test results and discussion

The BLSA technique is applied by using the proposed objective function to determine optimal RCS placement and sizing. The results of the proposed BLSA are compared with the results of GA and binary PSO (BPSO). For the same purpose, BLSA is also implemented by using the conventional objective function developed in [24] for the same road network and power system, and the results are compared with those of GA and BPSO. For a fair comparison, population size and maximum iteration are set as 80 and 50, respectively, in all cases. The BLSA algorithm-dependent parameter, i.e., channel time, is set as 10; the selection and mutation rates are set as 0.5 and 0.07, respectively, for GA; and c1, c2, minimum weight, and maximum weight are set as 1, 1, 0.2, and 0.9, respectively, for BPSO. The results of using this function are compared with the results of using the conventional function to evaluate the effectiveness of the proposed objective function in optimal RCS planning.

### Test results for optimal RCS placement and sizing

This section presents the optimization results of RCS placement and sizing by using the proposed and conventional objective functions. Table 3 shows the three objectives that consider possible combinations of sub-objective functions. The sign (√) that shows a particular sub-objective function is considered in the optimization problem, and the symbol (×) indicates that the sub-objective function is not considered in the problem. Table 4 provides the best optimization results of optimal RCS planning by using the proposed and conventional objective functions obtained using the BLSA, GA, and BPSO techniques. The shaded figure shown in Table 4 corresponds to the components that are not considered in the objective function during optimization. The results of using the proposed and conventional objective functions for the three objectives are summarized as follows.

**Table 3:** Three objectives of optimal RCS placement and sizing.

Objective function	Proposed objective function			Conventional Objective function		
	Transportation energy loss (TEL) cost	Build-up (BU) cost	Substation energy loss (SEL) cost	EV loss (EVL) cost	Station development & electrification (SDE) cost	Grid loss (GL) cost
Objective 1	√	√	√	√	√	√
Objective 2	√	√	×	√	√	×
Objective 3	×	√	√	×	√	√

**Table 4:** Optimization results of each objective using BLSA.

	Objective function	RCS location	Capacity of RCS (kW)	Power loss (kW)		TEL (pu)	BU (pu)	SEL (pu)	Total cost (pu)
				Without EV	With only EV				
Objective 1	Proposed	2,4,7,13,17,19	288,480,192,384,384, 288		69.98	0.1383	0.1891	0.0368	0.3642
								
	Conventional	1,2,4,7,9,10,11,12,18,20	192,96,192,192,288,192,192,192,288,288		43.43	0.1585	0.2240	0.0228	0.4053
Objective 2	Proposed	6,10,12,19	576,672,288,480	221.7	254.2	0.1458	0.1777	0.1375	0.4610
								
	Conventional	1,2,4,7,9,10,11,12,18,20	192,96,192,192,288,192,192, 192,288,288		43.43	0.1585	0.2240	0.0228	0.4053
Objective 3	Proposed	2,4,7,13,16,18	288,576,192,384,384,192		68.16	0.1439	0.1886	0.0362	0.3687
								
	Conventional	7	1920		26.17	0.7500	0.1650	0.0147	0.9297

#### Objective 1

**Fig 7:**
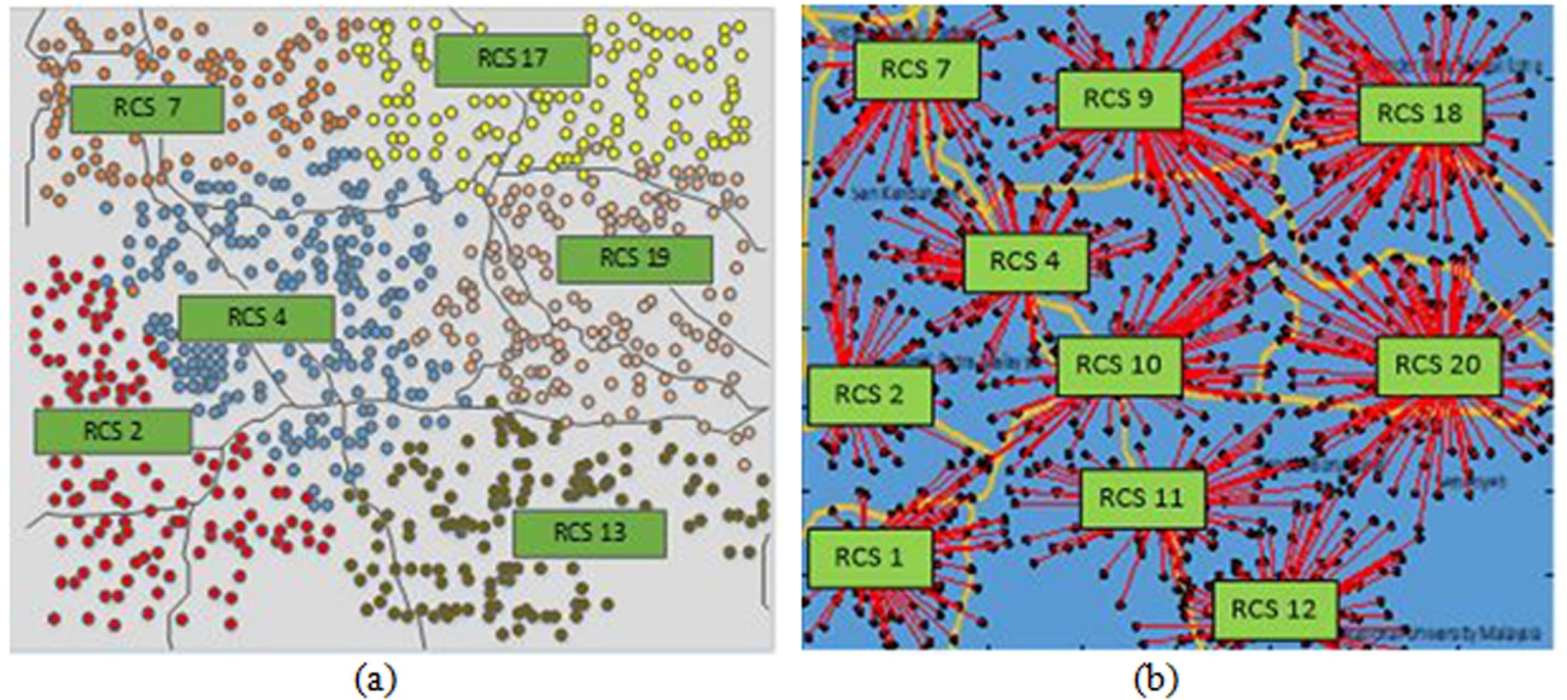
Optimal RCS locations of Objective 1. **(a)** Location of RCSs for Objective 1 using proposed objective function and **(b)** Location of RCSs for Objective 1 using conventional objective function.

In Objective 1, the desired objective function is to minimize BU, TEL, and SEL costs. The results of the proposed objective function in Table 4 show that BU cost provides 52% (0.1891 pu) of the total cost, whereas TEL and SEL costs constitute over 38% (0.1383 pu) and 10% (0.0368 pu) of the total cost, respectively. The BLSA techniques, using the proposed objective function, identify six RCSs to be installed at locations 2, 4, 7, 13, 17, and 19 based on the lowest fitness value to cover the entire area, as shown in Fig 7A. The balloon marker represents the EV positions, whereas the different colors characterize the group of EV locations to the nearest RCS based on time taken to reach an RCS. The corresponding station capacities are 288, 480, 192, 384, 384, and 288 kW, as shown in Table 4. This table also indicates that power losses without and with only EV loads are 221.7 kW and 69.98 kW, respectively. The BLSA techniques, using the conventional objective function, identify 10 RCSs, as shown in Fig 7B. In the figure, the blue points indicate the EV positions, whereas the red lines represent the Euclidean distance of EVs to the nearest RCS. The contributions of BU, TEL, and SEL costs to the total cost are 55%, 39%, and 6%, respectively. In the conventional objective function, the power loss due to only EV load is 43.43 kW because harmonic currents are not considered.

The total cost of using the proposed objective function decreases by over 10% compared with that using the conventional objective function, in which BU cost decreases by over 15% because of four less RCSs. The TEL cost decreases by over 12% in spite of the fewer number of RCSs than that using the conventional objective function.

#### Objective 2

**Fig 8:**
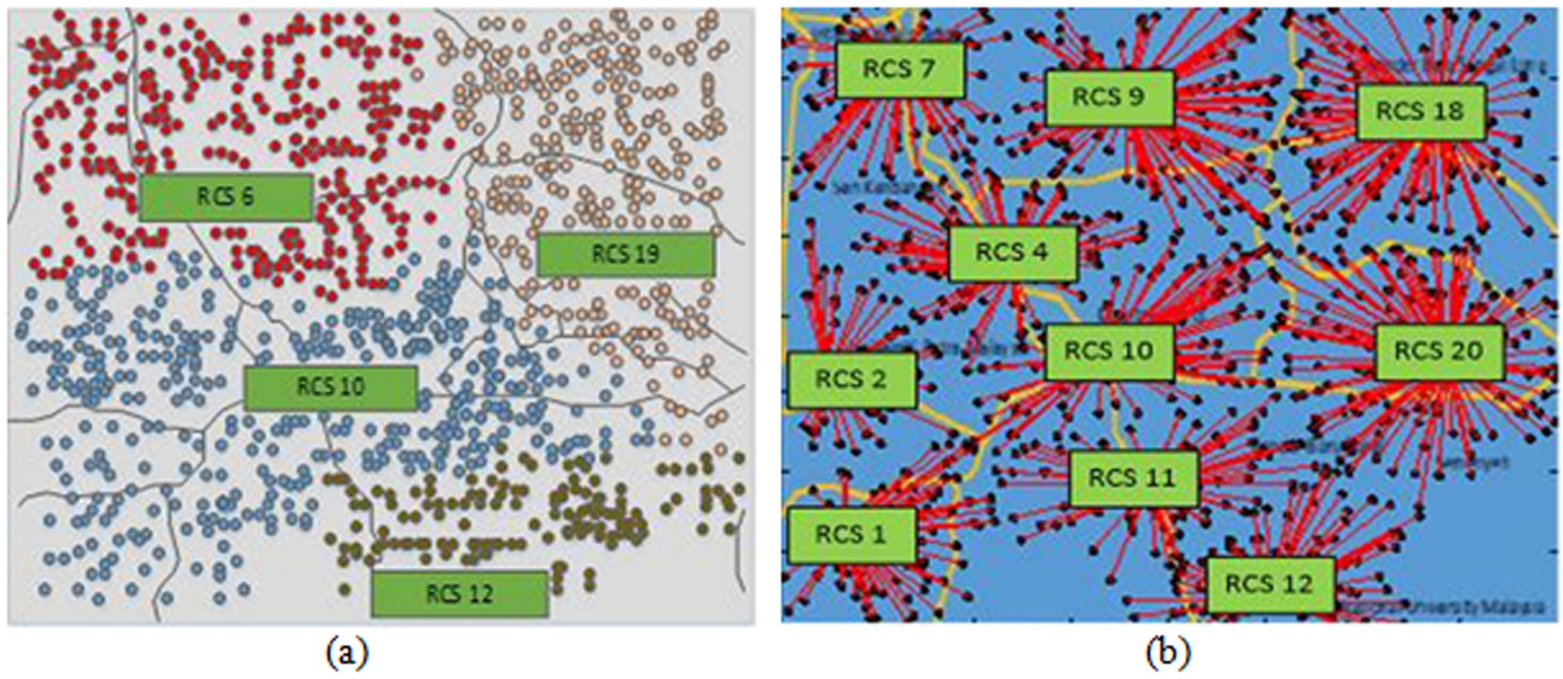
Optimal RCS locations of Objective 2. **(a)** Location of RCSs for Objective 2 using proposed objective function and **(b)** Location of RCSs for Objective 2 using conventional objective function.

Objective 2 assumes that the power system loss component is insignificant in RCS planning. The total cost of Objective 2 increases by more than 26% with SEL cost compared with the normalized results of the proposed objective function, as shown in Table 4. However, the number of RCSs decreases from 10 to 4, as shown in Fig 8A. Total power loss sharply increases from 291.68 kW to 475.9 kW, in which the loss incurred by only EV loads is 254.2 kW. Compared with Objective 1, Objective 2 presents exactly the same total cost as using the conventional objective function.

In Objective 2, the number of optimal RCSs is identified based on TEL and BU costs. The total costs of TEL and BU using the proposed objective function are reduced by 15%, in which TEL cost is reduced by 8% due to the fewer number of RCSs than that using the conventional objective function.

#### Objective 3

**Fig 9:**
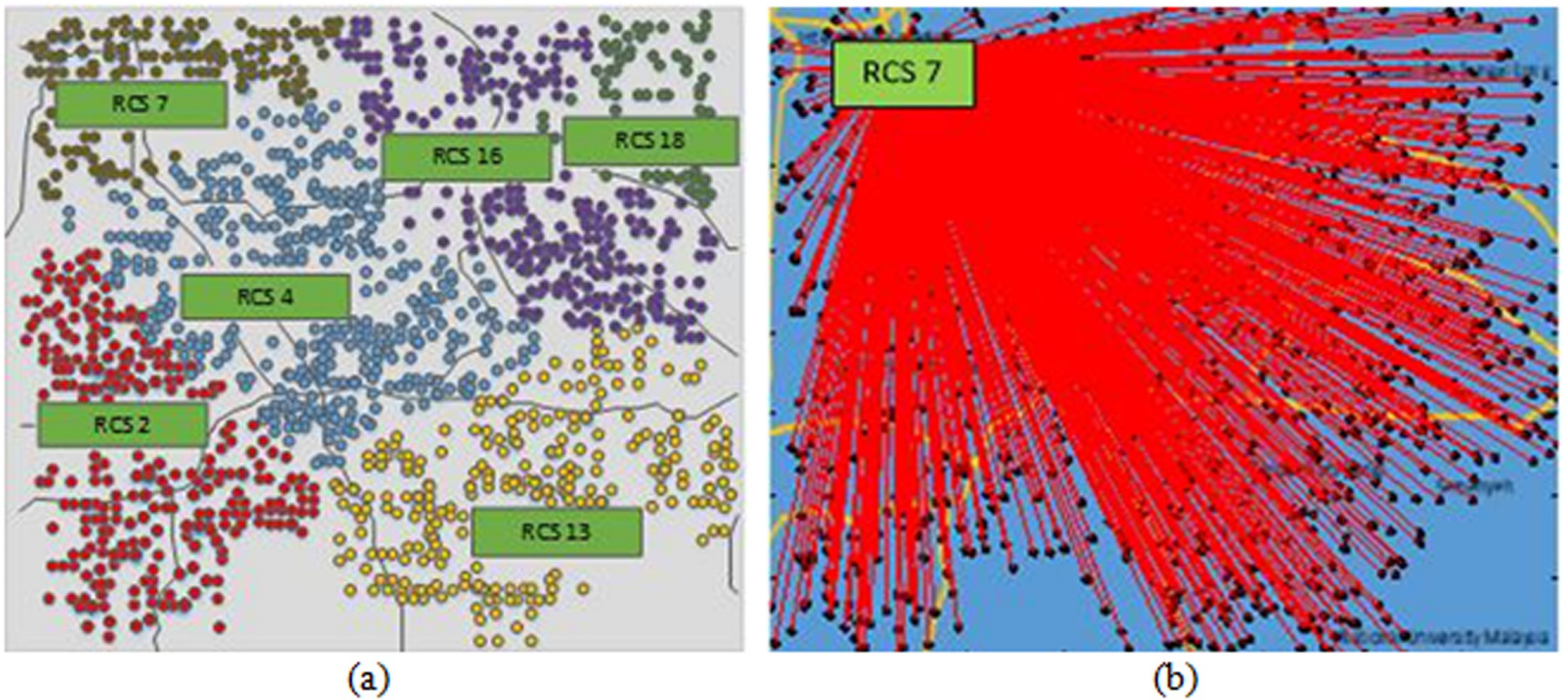
Optimal RCS locations of Objective 3. **(a)** Location of RCSs for Objective 3 using proposed objective function and **(b)** Location of RCSs for Objective 3 using conventional objective function.

The effect of TEL cost is disregarded in Objective 3. The comparison of the results of Objectives 3 and 1 shows that the same number of RCSs but slightly different RCS locations are identified by the BLSA techniques using the proposed objective function, as shown in Table 4 and Fig 9A. Only one large RCS is identified at optimal location 7 by using the conventional objective function, as shown in Fig 9B, with 1920 kW capacity.

The total costs of SEL and BU by using the conventional objective function decrease by 20% compared with those by using the proposed objective function because only one RCS is identified for the conventional objective function. Only one RCS is selected because EV SOC is not considered. In such case, an EV can reach any RCS location although it is far from the EV position. However, this assumption is considered unrealistic.

**Fig 10:**
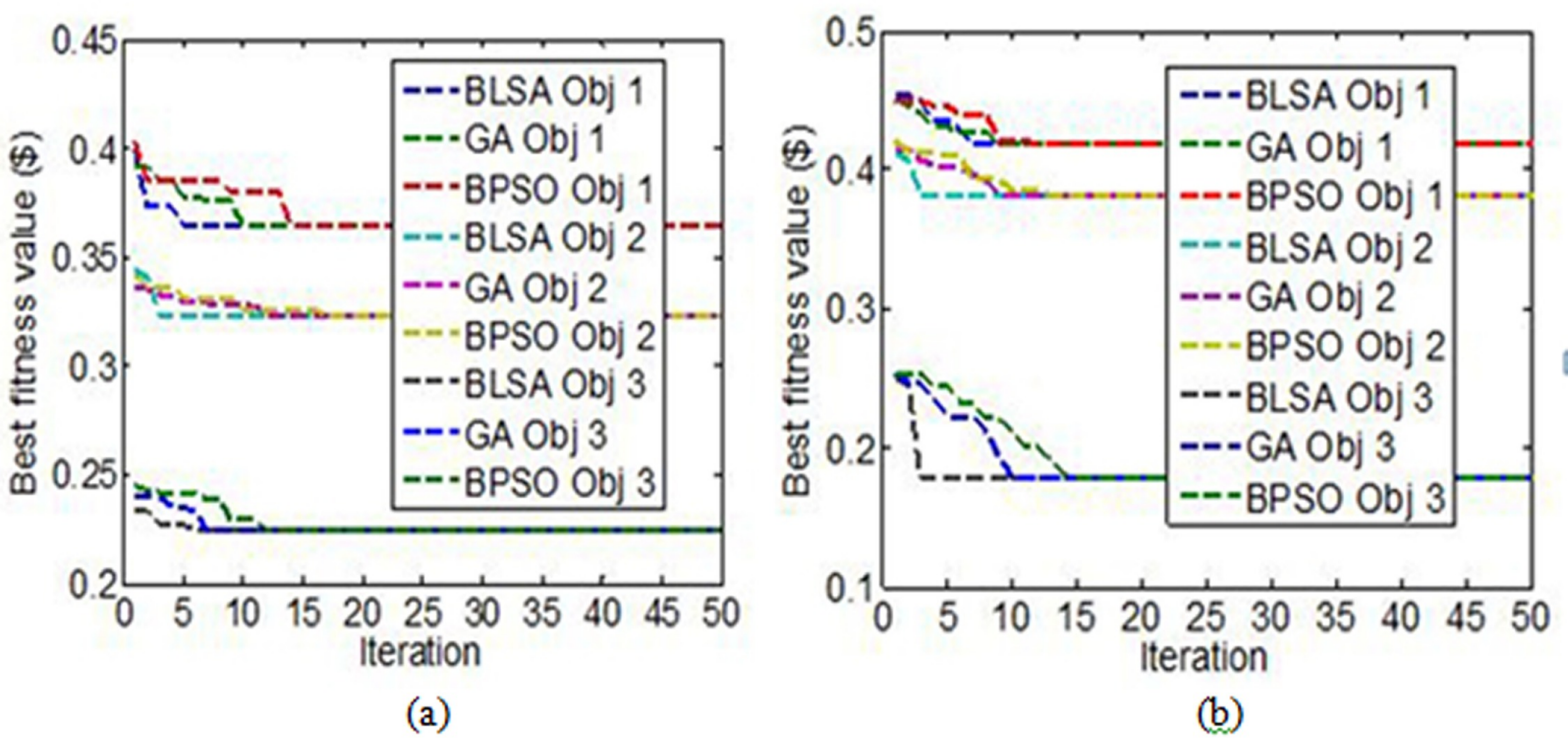
Characteristics of different optimization techniques. **(a)** Convergence characteristic curves of the BLSA, GA, and BPSO techniques in solving Objectives 1, 2, and 3 using proposed objective function and **(b)** Convergence characteristic curves of the BLSA, GA, and BPSO techniques in solving Objectives 1, 2, and 3 using conventional objective function.

Table 5 shows the performance of the BLSA, GA, and BPSO techniques in determining the optimal placement and sizing of RCS in relation to fitness value, optimized number of RCS, consistency of results, and minimum number of iterations in which the optimization results start to converge with the best fitness value. All the optimization techniques are executed 30 independent times by using the proposed and conventional objective functions for each objective. The performance of these optimization techniques is also depicted in Fig 10A and 10B.

**Table 5:** Performance of BLSA, GA, and BPSO in obtaining the optimal RCS placement and sizing solution.

	Objective function	Parameters	Best Results		
			BLSA	GA	BPSO
Objective 1	Proposed	Fitness value	0.3642	0.3642	0.3642
		No. of RCS	6	6	6
		Consistency (%)	93.3	90	13.3
		Iteration	5	10	14
		Comput. time (s)	1173.43	1301.71	1380.94
				
	Conventional	Fitness value	0.4185	0.4185	0.4185
		No. of RCS	10	10	10
		Consistency (%)	86.6	83.3	30
		Iteration	7	9	12
		Comput. time (s)	1629.95	1746.74	1692.09
Objective 2	Proposed	Fitness value	0.3235	0.3235	0.3235
		No. of RCS	4	4	4
		Consistency (%)	100	100	23.3
		Iteration	3	12	17
		Comput. time (s)	1130.60	1130.92	1259.54
				
	Conventional	Fitness value	0.3825	0.3825	0.3825
		No. of RCS	10	10	10
		Consistency (%)	100	100	46.6
		Iteration	3	9	13
		Comput. time (s)	1674.83	1764.76	1754.22
Objective 3	Proposed	Fitness value	0.2248	0.2248	0.2248
		No. of RCS	6	6	6
		Consistency (%)	100	96.6	40
		Iteration	6	7	12
		Comput. time (s)	1089.20	1108.34	1418.38
				
	Conventional	Fitness value	0.1797	0.1797	0.1797
		No. of RCS	1	1	1
		Consistency (%)	100	100	50
		Iteration	3	10	15
		Comput. time (s)	872.50	886.81	912.23

Table 5 indicates that the BLSA, GA, and BPSO techniques can determine the best fitness value for Objective 1 by using the proposed and conventional objective functions. However, the BSLA technique (93.3% and 86.6%) is more consistent than the GA technique (90% and 83.3%) and the BPSO technique (13.3% and 30%). The BLSA requires less computational times and exactly half and one-third iterations compared with those of GA and BPSO, respectively, to determine the best fitness value for the proposed objective function, as shown in Fig 10A and Table 5. BLSA also requires less number of iterations and computational time (7 iterations and 1629.95 s) than those of GA (9 iterations and 1746.74 s) and BPSO (12 iterations and 1692.09 s) for the conventional objective function, as shown in Fig 10B and Table 5.

For the results of Objectives 2 and 3 shown in Table 5 and Fig 10A and 10B, BLSA is more consistent and requires less number of iterations and computational times than GA and BPSO by using the proposed and conventional objective functions.

**Fig 11:**
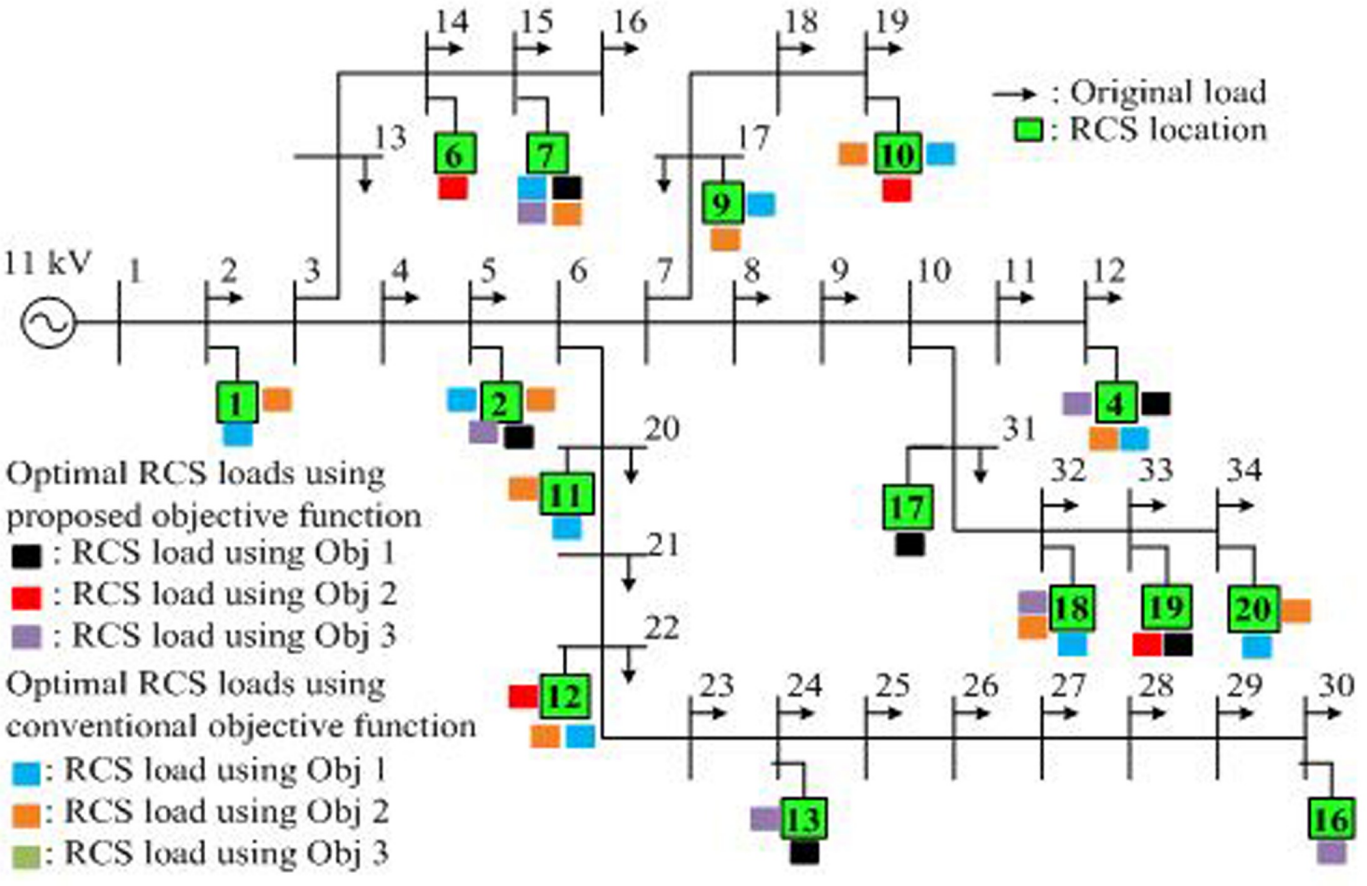
RCS locations for two methods. Optimal RCS loads for Objectives 1, 2, and 3 using the proposed and conventional objective functions.

The optimal placements of RCSs connected in the IEEE 34-bus test system for each objective using the proposed and conventional objective functions are shown in Fig 11. The green rectangle indicates the RCS optimal location number, as shown earlier in Fig 6. The black, red, and gray rectangles represent the optimal RCS locations obtained using the proposed objective function, whereas the blue, orange, and olive green rectangles represent the optimal RCS locations obtained using the conventional objective function for Objectives 1, 2, and 3, respectively.

## Discussion

The comparison between the total costs of the proposed and conventional objective functions shows that the total cost of Objective 1 for both objective functions is less than those of all the other objectives. In Objective 1, however, the total cost of using the proposed objective function decreases by over 10% compared with that of using the conventional objective function. Therefore, Objective 1 of the proposed objective function can be utilized by decision makers to consider and minimize all costs that arise from different situations. Objectives 2 and 3 are unrealistic. For Objective 2, SEL cost as a result of enormous power loss during rapid charging is inevitable. For Objective 3, EV users need to travel a long distance and pay high recharging fees. Hence, TEL and SEL costs should be considered in RCS planning for the convenience of EV users and RCS developers.

## Conclusion

This study suggests a novel approach for optimal RCS placement and sizing by considering Google Maps API, battery SOC, traffic density, and harmonic load flow. The optimization problem is formulated by relying on three cost functions (TEL, BU, and SEL) and is solved by utilizing the BLSA technique. The optimization technique is also applied by using the conventional objective function under the same road and power networks. The results of the proposed technique using the proposed and conventional objective functions are compared with the results of GA and BPSO. The outcomes of the proposed objective function are compared with the outcomes of the conventional objective function. The case study shows that the proposed optimization technique and the proposed objective function can be beneficial for EV users and RCS developers in determining optimal RCS placement and sizing with minimum total cost and without stressing the power grid.

## Supporting information

S1 File **Table A.** Electrical vehicles (EVs) location addresses. **Table B.** Rapid charging stations (RCSs) location addresses.
(DOCX)
