# Supplementary material for: Improved approach for electric vehicle rapid charging station placement and sizing using Google maps and binary lightning search algorithm
Source: PLoS One. 2017 Dec 8;12(12):e0189170. doi: 10.1371/journal.pone.0189170 (PMC5722383; doi:10.1371/journal.pone.0189170)
Supplement: S1 File — Table A. Electrical vehicles (EVs) location addresses. Table B. Rapid charging stations (RCSs) location addresses. (DOCX) [file pone.0189170.s001.docx]

**S1 Table A.** Electrical vehicles (EVs) location addresses

| **Latitude** | **Longitude** | **Latitude** | **Longitude** | **Latitude** | **Longitude** | **Latitude** | **Longitude** |
| --- | --- | --- | --- | --- | --- | --- | --- |
| 2.9486 | 101.7584 | 2.926 | 101.7618 | 2.9429 | 101.759 | 2.9678 | 101.7484 |
| 2.9513 | 101.7766 | 2.9377 | 101.7491 | 2.9387 | 101.7492 | 2.9761 | 101.7684 |
| 2.9287 | 101.7672 | 2.9361 | 101.7816 | 2.9751 | 101.7347 | 2.9755 | 101.7409 |
| 2.9515 | 101.7659 | 2.9472 | 101.7452 | 2.9645 | 101.7444 | 2.9714 | 101.7317 |
| 2.9433 | 101.7799 | 2.9481 | 101.7744 | 2.9605 | 101.7741 | 2.9642 | 101.7709 |
| 2.9278 | 101.7559 | 2.9304 | 101.7761 | 2.9649 | 101.7316 | 2.9759 | 101.7575 |
| 2.9331 | 101.7738 | 2.9392 | 101.778 | 2.9566 | 101.7811 | 2.9684 | 101.7564 |
| 2.9409 | 101.7736 | 2.9379 | 101.7482 | 2.9576 | 101.7702 | 2.9635 | 101.7798 |
| 2.9528 | 101.7595 | 2.9437 | 101.7602 | 2.9794 | 101.7569 | 2.9794 | 101.7635 |
| 2.953 | 101.7666 | 2.9456 | 101.7549 | 2.9798 | 101.7618 | 2.9777 | 101.764 |
| 2.9296 | 101.7479 | 2.9469 | 101.7754 | 2.9695 | 101.7431 | 2.9689 | 101.7773 |
| 2.9531 | 101.7471 | 2.933 | 101.7614 | 2.9556 | 101.7552 | 2.9708 | 101.7743 |
| 2.9528 | 101.7652 | 2.9447 | 101.7796 | 2.9603 | 101.783 | 2.9699 | 101.7617 |
| 2.9391 | 101.7746 | 2.944 | 101.7519 | 2.9635 | 101.7601 | 2.9596 | 101.7401 |
| 2.9482 | 101.7805 | 2.9297 | 101.755 | 2.9762 | 101.7587 | 2.9621 | 101.7432 |
| 2.9291 | 101.7499 | 2.9285 | 101.7505 | 2.9544 | 101.7427 | 2.9667 | 101.7788 |
| 2.9372 | 101.7666 | 2.9395 | 101.7502 | 2.9552 | 101.7569 | 2.9602 | 101.7316 |
| 2.9516 | 101.7628 | 2.9528 | 101.778 | 2.9586 | 101.7643 | 2.9768 | 101.7569 |
| 2.948 | 101.7455 | 2.9349 | 101.767 | 2.9715 | 101.7674 | 2.9593 | 101.7392 |
| 2.9528 | 101.7578 | 2.942 | 101.7659 | 2.9738 | 101.7518 | 2.9601 | 101.7838 |
| 2.944 | 101.7512 | 2.9315 | 101.7505 | 2.9715 | 101.7502 | 2.9586 | 101.7692 |
| 2.926 | 101.7752 | 2.9468 | 101.7774 | 2.9662 | 101.7843 | 2.9601 | 101.7575 |
| 2.9496 | 101.7568 | 2.9324 | 101.7686 | 2.9688 | 101.7321 | 2.9658 | 101.7559 |
| 2.9521 | 101.7651 | 2.9397 | 101.7583 | 2.962 | 101.7787 | 2.9624 | 101.7333 |
| 2.9447 | 101.7513 | 2.9453 | 101.7645 | 2.9741 | 101.7802 | 2.9789 | 101.7675 |
| 2.947 | 101.7679 | 2.9508 | 101.7603 | 2.9591 | 101.7738 | 2.9656 | 101.7323 |
| 2.9466 | 101.755 | 2.9528 | 101.7479 | 2.9725 | 101.7354 | 2.959 | 101.7339 |
| 2.9364 | 101.7699 | 2.9409 | 101.7541 | 2.959 | 101.7444 | 2.9784 | 101.7587 |
| 2.944 | 101.7712 | 2.929 | 101.7497 | 2.9639 | 101.7484 | 2.9805 | 101.7353 |
| 2.93 | 101.7734 | 2.9293 | 101.752 | 2.9709 | 101.7674 | 2.9658 | 101.775 |
| 2.9455 | 101.7621 | 2.9325 | 101.7541 | 2.9751 | 101.7375 | 2.957 | 101.775 |
| 2.9259 | 101.7482 | 2.9494 | 101.7609 | 2.9562 | 101.7697 | 2.961 | 101.7697 |
| 2.933 | 101.7537 | 2.9324 | 101.7469 | 2.9791 | 101.7359 | 2.965 | 101.7382 |
| 2.9263 | 101.7797 | 2.9486 | 101.7793 | 2.9749 | 101.766 | 2.9701 | 101.7663 |
| 2.9278 | 101.7508 | 2.9321 | 101.7809 | 2.9671 | 101.7572 | 2.9611 | 101.7585 |
| 2.9489 | 101.7764 | 2.9519 | 101.7637 | 2.9658 | 101.7728 | 2.9703 | 101.7835 |
| 2.9452 | 101.7655 | 2.9351 | 101.7636 | 2.9661 | 101.7693 | 2.9732 | 101.7657 |
| 2.9342 | 101.7829 | 2.9307 | 101.7578 | 2.9623 | 101.7797 | 2.96 | 101.774 |
| 2.9526 | 101.748 | 2.9323 | 101.7792 | 2.9677 | 101.779 | 2.9572 | 101.755 |
| **Latitude** | **Longitude** | **Latitude** | **Longitude** | **Latitude** | **Longitude** | **Latitude** | **Longitude** |
| 2.962 | 101.7538 | 2.9332 | 101.6842 | 2.9446 | 101.7091 | 2.982 | 101.7124 |
| 2.9626 | 101.7754 | 2.9309 | 101.7083 | 2.9394 | 101.7004 | 2.9784 | 101.7156 |
| 2.9655 | 101.7346 | 2.971 | 101.7098 | 2.9324 | 101.6928 | 2.9785 | 101.723 |
| 2.9677 | 101.7373 | 2.9329 | 101.6703 | 2.9332 | 101.7066 | 2.977 | 101.7272 |
| 2.933 | 101.6926 | 2.9265 | 101.6821 | 2.9957 | 101.7066 | 2.9879 | 101.7174 |
| 2.943 | 101.6719 | 2.9508 | 101.6801 | 2.9967 | 101.6861 | 2.9893 | 101.7087 |
| 2.9632 | 101.7037 | 2.9656 | 101.7081 | 2.9953 | 101.6871 | 2.9867 | 101.7247 |
| 2.962 | 101.6957 | 2.9558 | 101.7192 | 3.0052 | 101.6883 | 2.9994 | 101.7087 |
| 2.9278 | 101.7098 | 2.9338 | 101.6877 | 2.9817 | 101.7132 | 2.9951 | 101.6828 |
| 2.9434 | 101.7101 | 2.942 | 101.7152 | 2.9977 | 101.7048 | 2.9997 | 101.6876 |
| 2.9492 | 101.7062 | 2.9462 | 101.7085 | 2.9826 | 101.6907 | 3.0049 | 101.7217 |
| 2.9442 | 101.6681 | 2.9702 | 101.6664 | 2.9788 | 101.7048 | 3.0061 | 101.7043 |
| 2.9552 | 101.6703 | 2.9322 | 101.7039 | 2.9944 | 101.6888 | 2.9811 | 101.7209 |
| 2.9539 | 101.6861 | 2.9644 | 101.6904 | 2.9894 | 101.6845 | 2.9794 | 101.6916 |
| 2.9384 | 101.6994 | 2.9547 | 101.7237 | 2.9897 | 101.7211 | 2.9973 | 101.7074 |
| 2.9449 | 101.7072 | 2.9423 | 101.6661 | 2.9962 | 101.7078 | 2.978 | 101.711 |
| 2.9257 | 101.6917 | 2.9338 | 101.6951 | 2.9996 | 101.7248 | 2.9918 | 101.6835 |
| 2.9703 | 101.7177 | 2.9447 | 101.6927 | 2.9862 | 101.714 | 2.992 | 101.7103 |
| 2.9327 | 101.7113 | 2.9472 | 101.695 | 2.9962 | 101.7088 | 3.0026 | 101.6987 |
| 2.9299 | 101.727 | 2.9305 | 101.7145 | 2.9883 | 101.7195 | 2.9905 | 101.6843 |
| 2.9421 | 101.6995 | 2.9521 | 101.6863 | 3.0019 | 101.7224 | 2.9876 | 101.7045 |
| 2.9341 | 101.6865 | 2.9354 | 101.7154 | 3.0017 | 101.7275 | 2.9965 | 101.6909 |
| 2.9475 | 101.6727 | 2.9427 | 101.6957 | 2.9832 | 101.682 | 2.9987 | 101.6877 |
| 2.9406 | 101.7045 | 2.9518 | 101.6683 | 2.9946 | 101.7218 | 2.9916 | 101.6915 |
| 2.9688 | 101.7151 | 2.9366 | 101.6771 | 2.9936 | 101.7102 | 2.9861 | 101.6887 |
| 2.9673 | 101.6927 | 2.9384 | 101.7115 | 2.9923 | 101.7275 | 2.9798 | 101.6907 |
| 2.9274 | 101.6717 | 2.9534 | 101.6958 | 3.0028 | 101.7063 | 2.9938 | 101.684 |
| 2.9589 | 101.6828 | 2.9372 | 101.6756 | 2.9835 | 101.7041 | 2.9834 | 101.7112 |
| 2.9374 | 101.6757 | 2.9629 | 101.6875 | 2.9852 | 101.7189 | 2.9764 | 101.695 |
| 2.9445 | 101.6837 | 2.9702 | 101.7043 | 2.9788 | 101.6925 | 2.9992 | 101.7068 |
| 2.9502 | 101.6937 | 2.9586 | 101.6781 | 3.0051 | 101.7049 | 2.9828 | 101.714 |
| 2.9684 | 101.6992 | 2.9408 | 101.7125 | 2.9957 | 101.7234 | 2.9892 | 101.705 |
| 2.9442 | 101.6948 | 2.9519 | 101.6813 | 2.9903 | 101.7084 | 2.997 | 101.7066 |
| 2.9702 | 101.7211 | 2.93 | 101.7238 | 2.9955 | 101.7209 | 2.9865 | 101.7025 |
| 2.9389 | 101.6986 | 2.9667 | 101.683 | 2.9924 | 101.716 | 2.9986 | 101.6877 |
| 2.9573 | 101.7254 | 2.9655 | 101.7142 | 2.9957 | 101.709 | 2.9876 | 101.7046 |
| 2.9557 | 101.7062 | 2.9626 | 101.6779 | 2.9924 | 101.6933 | 2.9969 | 101.7212 |
| 2.9498 | 101.7263 | 2.937 | 101.6841 | 2.9981 | 101.7127 | 2.9975 | 101.7222 |
| 2.9571 | 101.6812 | 2.9523 | 101.6717 | 2.9917 | 101.6858 | 2.9892 | 101.6944 |
| 2.9557 | 101.7086 | 2.926 | 101.7023 | 3.0068 | 101.7108 | 2.9756 | 101.6916 |
| **Latitude** | **Longitude** | **Latitude** | **Longitude** | **Latitude** | **Longitude** | **Latitude** | **Longitude** |
| 2.9856 | 101.708 | 3.0252 | 101.7218 | 3.0327 | 101.6902 | 3.0235 | 101.7334 |
| 2.9886 | 101.7115 | 3.0146 | 101.6889 | 3.0103 | 101.7294 | 3.0277 | 101.7041 |
| 2.9836 | 101.7012 | 3.0328 | 101.7124 | 3.007 | 101.6915 | 3.0074 | 101.7218 |
| 2.9813 | 101.6915 | 3.0078 | 101.6899 | 3.0138 | 101.6921 | 3.0081 | 101.7444 |
| 3.0013 | 101.7256 | 3.0356 | 101.6904 | 3.0186 | 101.6894 | 3.0216 | 101.7268 |
| 2.9888 | 101.6858 | 3.0213 | 101.7408 | 3.0073 | 101.6927 | 3.0195 | 101.7071 |
| 3.0034 | 101.6869 | 3.0266 | 101.7039 | 3.0359 | 101.6946 | 3.0325 | 101.7044 |
| 2.9875 | 101.6885 | 3.036 | 101.7273 | 3.0146 | 101.6967 | 3.0296 | 101.7159 |
| 2.9996 | 101.6897 | 3.0132 | 101.7543 | 3.0135 | 101.7058 | 3.0275 | 101.7137 |
| 2.9877 | 101.7106 | 3.0173 | 101.7144 | 3.006 | 101.7057 | 3.0056 | 101.709 |
| 3.0009 | 101.7084 | 3.0189 | 101.7341 | 3.0135 | 101.6983 | 3.0063 | 101.7239 |
| 2.9992 | 101.6844 | 3.0284 | 101.7389 | 3.0055 | 101.7008 | 3.0068 | 101.7377 |
| 2.9871 | 101.7248 | 3.0302 | 101.7144 | 3.0202 | 101.749 | 3.0295 | 101.7138 |
| 2.9819 | 101.7155 | 3.0072 | 101.7312 | 3.0284 | 101.7347 | 3.0342 | 101.7142 |
| 3.0003 | 101.7159 | 3.0097 | 101.6902 | 3.0242 | 101.7237 | 3.0259 | 101.6914 |
| 3.0054 | 101.6849 | 3.0155 | 101.752 | 3.0069 | 101.6958 | 3.0082 | 101.6838 |
| 2.9855 | 101.7216 | 3.0058 | 101.6961 | 3.0066 | 101.6979 | 3.0271 | 101.7038 |
| 2.9965 | 101.725 | 3.0207 | 101.702 | 3.0289 | 101.6878 | 3.0075 | 101.7058 |
| 2.989 | 101.7273 | 3.0147 | 101.7418 | 3.033 | 101.7505 | 3.0078 | 101.731 |
| 3.0017 | 101.7215 | 3.0096 | 101.7186 | 3.0211 | 101.735 | 3.0245 | 101.7538 |
| 2.9996 | 101.7181 | 3.0107 | 101.7397 | 3.0075 | 101.7238 | 3.0145 | 101.7522 |
| 2.9804 | 101.7056 | 3.033 | 101.7117 | 3.0304 | 101.7055 | 3.0249 | 101.7163 |
| 3.0026 | 101.6902 | 3.0256 | 101.7025 | 3.0148 | 101.6945 | 3.028 | 101.7 |
| 3.0067 | 101.7003 | 3.019 | 101.6848 | 3.0134 | 101.7287 | 3.0227 | 101.7393 |
| 2.9915 | 101.6882 | 3.0332 | 101.7325 | 3.0279 | 101.7561 | 3.0277 | 101.7389 |
| 3.0033 | 101.6834 | 3.0073 | 101.7142 | 3.0043 | 101.6948 | 3.0115 | 101.7375 |
| 2.9938 | 101.7252 | 3.0279 | 101.7159 | 3.0056 | 101.7013 | 3.0275 | 101.7378 |
| 2.98 | 101.6959 | 3.0276 | 101.7277 | 3.0254 | 101.7118 | 3.0351 | 101.6899 |
| 2.9814 | 101.6956 | 3.022 | 101.6865 | 3.0233 | 101.6875 | 3.0317 | 101.7331 |
| 2.988 | 101.6973 | 3.0099 | 101.7057 | 3.0208 | 101.7333 | 3.0068 | 101.7167 |
| 2.999 | 101.7035 | 3.0231 | 101.74 | 3.0274 | 101.7122 | 3.0157 | 101.6979 |
| 3.0014 | 101.7118 | 3.0136 | 101.7342 | 3.0266 | 101.7557 | 3.0158 | 101.6894 |
| 3.0003 | 101.6832 | 3.0083 | 101.6914 | 3.029 | 101.7122 | 3.0259 | 101.7438 |
| 2.9852 | 101.7207 | 3.0108 | 101.6918 | 3.0132 | 101.7286 | 3.0231 | 101.6951 |
| 3.0219 | 101.7183 | 3.0326 | 101.6889 | 3.0262 | 101.6936 | 3.0293 | 101.6943 |
| 3.0313 | 101.6934 | 3.0063 | 101.6826 | 3.0218 | 101.7106 | 3.0158 | 101.7319 |
| 3.0151 | 101.7406 | 3.0118 | 101.7137 | 3.0167 | 101.6941 | 3.0106 | 101.7491 |
| 3.0183 | 101.6895 | 3.0057 | 101.7312 | 3.006 | 101.7389 | 3.0068 | 101.7207 |
| 3.0057 | 101.7041 | 3.0181 | 101.7362 | 3.029 | 101.7473 | 3.0287 | 101.7347 |
| 3.0097 | 101.6998 | 3.0044 | 101.7218 | 3.0148 | 101.7083 | 3.0106 | 101.6935 |
| **Latitude** | **Longitude** | **Latitude** | **Longitude** | **Latitude** | **Longitude** | **Latitude** | **Longitude** |
| 3.0164 | 101.7535 | 3.0647 | 101.6988 | 3.0493 | 101.6911 | 3.0456 | 101.7169 |
| 3.0217 | 101.7226 | 3.051 | 101.7205 | 3.0557 | 101.717 | 3.047 | 101.6975 |
| 3.0113 | 101.733 | 3.0406 | 101.7668 | 3.0545 | 101.7258 | 3.0574 | 101.763 |
| 3.0245 | 101.6847 | 3.0624 | 101.7659 | 3.0533 | 101.7077 | 3.0533 | 101.6951 |
| 3.0608 | 101.7063 | 3.056 | 101.7484 | 3.0464 | 101.7485 | 3.0501 | 101.767 |
| 3.0592 | 101.7101 | 3.0486 | 101.7402 | 3.0457 | 101.7606 | 3.0553 | 101.7213 |
| 3.0422 | 101.7449 | 3.0659 | 101.7165 | 3.0512 | 101.7112 | 3.0593 | 101.6911 |
| 3.0532 | 101.7281 | 3.0451 | 101.7675 | 3.0451 | 101.7499 | 3.0548 | 101.7164 |
| 3.0478 | 101.7406 | 3.0566 | 101.6977 | 3.0607 | 101.6988 | 3.0539 | 101.7503 |
| 3.0538 | 101.7073 | 3.0553 | 101.7498 | 3.0656 | 101.759 | 3.0548 | 101.7553 |
| 3.0498 | 101.7022 | 3.0495 | 101.7426 | 3.0398 | 101.6989 | 3.0528 | 101.7339 |
| 3.0502 | 101.7583 | 3.0428 | 101.7587 | 3.0535 | 101.7376 | 3.0412 | 101.746 |
| 3.0439 | 101.753 | 3.0397 | 101.7213 | 3.0414 | 101.7185 | 3.0584 | 101.7639 |
| 3.0459 | 101.7674 | 3.0504 | 101.7515 | 3.0607 | 101.7564 | 3.0659 | 101.6917 |
| 3.0396 | 101.6963 | 3.044 | 101.7588 | 3.0657 | 101.7303 | 3.0486 | 101.7131 |
| 3.0639 | 101.7027 | 3.0586 | 101.7147 | 3.0408 | 101.7291 | 3.0652 | 101.691 |
| 3.0566 | 101.6955 | 3.049 | 101.7345 | 3.0644 | 101.7624 | 3.0484 | 101.7038 |
| 3.0642 | 101.7291 | 3.0617 | 101.7712 | 3.0395 | 101.7174 | 3.0629 | 101.7489 |
| 3.0434 | 101.7036 | 3.0588 | 101.7342 | 3.0575 | 101.7257 | 3.0513 | 101.7491 |
| 3.0639 | 101.764 | 3.0544 | 101.7154 | 3.0602 | 101.7699 | 3.0502 | 101.7625 |
| 3.0605 | 101.6955 | 3.0438 | 101.7403 | 3.0534 | 101.6906 | 3.0449 | 101.7371 |
| 3.0546 | 101.6908 | 3.0648 | 101.718 | 3.0629 | 101.7707 | 3.0424 | 101.6931 |
| 3.0509 | 101.7349 | 3.0462 | 101.7521 | 3.0633 | 101.7033 | 3.0473 | 101.7664 |
| 3.046 | 101.7534 | 3.064 | 101.7226 | 3.0559 | 101.7444 | 3.0586 | 101.7558 |
| 3.0593 | 101.7138 | 3.045 | 101.7293 | 3.0427 | 101.7374 | 3.0601 | 101.7116 |
| 3.0452 | 101.7024 | 3.0491 | 101.7467 | 3.0449 | 101.7451 | 3.0577 | 101.7338 |
| 3.0407 | 101.7162 | 3.0414 | 101.7707 | 3.0439 | 101.718 | 3.0393 | 101.7717 |
| 3.0597 | 101.7051 | 3.0563 | 101.7152 | 3.0401 | 101.7403 | 3.0618 | 101.7485 |
| 3.0571 | 101.7309 | 3.0439 | 101.7591 | 3.0419 | 101.7568 | 3.0639 | 101.7592 |
| 3.0583 | 101.7649 | 3.0402 | 101.7506 | 3.0556 | 101.6887 | 3.0598 | 101.7243 |
| 3.0563 | 101.7411 | 3.0585 | 101.7691 | 3.0644 | 101.6942 | 3.0402 | 101.7275 |
| 3.0503 | 101.6957 | 3.0484 | 101.6897 | 3.0486 | 101.7708 | 3.0492 | 101.7352 |
| 3.0496 | 101.7206 | 3.0568 | 101.7177 | 3.0501 | 101.743 | 3.058 | 101.7101 |
| 3.061 | 101.6917 | 3.0494 | 101.744 | 3.0656 | 101.7069 | 3.0587 | 101.7514 |
| 3.0476 | 101.7301 | 3.0559 | 101.7112 | 3.0645 | 101.7217 | 2.9522 | 101.8179 |
| 3.061 | 101.7241 | 3.0396 | 101.7068 | 3.0573 | 101.6975 | 2.9585 | 101.8051 |
| 3.0603 | 101.7728 | 3.0636 | 101.7482 | 3.0657 | 101.7101 | 2.9435 | 101.8066 |
| 3.062 | 101.7568 | 3.0606 | 101.7407 | 3.0597 | 101.7092 | 2.9361 | 101.7904 |
| 3.0527 | 101.7288 | 3.0591 | 101.7378 | 3.0481 | 101.7155 | 2.9509 | 101.8174 |
| 3.0562 | 101.7639 | 3.061 | 101.7438 | 3.0569 | 101.7001 | 2.9459 | 101.8012 |
| **Latitude** | **Longitude** | **Latitude** | **Longitude** | **Latitude** | **Longitude** | **Latitude** | **Longitude** |
| 2.9647 | 101.7924 | 2.9484 | 101.8087 | 3.0009 | 101.7404 | 3.0532 | 101.7903 |
| 2.9643 | 101.8174 | 2.9728 | 101.8192 | 3.0063 | 101.7688 | 3.0175 | 101.8052 |
| 2.9594 | 101.8125 | 2.9735 | 101.811 | 2.9988 | 101.7408 | 3.0164 | 101.7718 |
| 2.9359 | 101.8168 | 2.968 | 101.7994 | 2.9903 | 101.7551 | 3.0139 | 101.8145 |
| 2.9309 | 101.7953 | 2.9471 | 101.8149 | 2.9968 | 101.742 | 2.9956 | 101.8059 |
| 2.9546 | 101.8092 | 2.95 | 101.7898 | 2.9961 | 101.7566 | 3.0056 | 101.7963 |
| 2.9432 | 101.8089 | 2.9409 | 101.7872 | 3.0101 | 101.7372 | 2.9912 | 101.7786 |
| 2.9713 | 101.7894 | 2.9645 | 101.788 | 3.0011 | 101.7638 | 3.0196 | 101.7934 |
| 2.9732 | 101.7997 | 2.9688 | 101.7909 | 3.0031 | 101.7667 | 3.0054 | 101.7943 |
| 2.9426 | 101.7949 | 2.9934 | 101.7521 | 3.0037 | 101.7691 | 3.0087 | 101.8167 |
| 2.9652 | 101.8108 | 2.9928 | 101.7613 | 2.994 | 101.7715 | 3.0193 | 101.8102 |
| 2.9694 | 101.7952 | 2.9853 | 101.7701 | 2.9984 | 101.7547 | 2.9939 | 101.8152 |
| 2.9563 | 101.8173 | 2.999 | 101.7644 | 2.9995 | 101.7465 | 3.0251 | 101.8019 |
| 2.9689 | 101.8148 | 2.9875 | 101.7617 | 2.9891 | 101.7405 | 3.0426 | 101.789 |
| 2.9715 | 101.799 | 2.9888 | 101.7408 | 2.9996 | 101.7548 | 3.0042 | 101.8139 |
| 2.9542 | 101.8029 | 3.0014 | 101.7506 | 3.0031 | 101.7575 | 3.0492 | 101.7925 |
| 2.962 | 101.81 | 3.0073 | 101.7577 | 2.9961 | 101.7637 | 2.9901 | 101.7809 |
| 2.9554 | 101.815 | 3.0103 | 101.7531 | 3.0067 | 101.7399 | 3.0167 | 101.7886 |
| 2.9311 | 101.8069 | 2.9998 | 101.7388 | 3.004 | 101.7602 | 2.9843 | 101.8031 |
| 2.9496 | 101.8057 | 3.0109 | 101.745 | 2.9944 | 101.7551 | 3.0023 | 101.7963 |
| 2.9584 | 101.7967 | 2.9994 | 101.7662 | 2.9968 | 101.743 | 2.9841 | 101.8056 |
| 2.9529 | 101.8014 | 2.9984 | 101.7375 | 2.995 | 101.7698 | 2.9997 | 101.8168 |
| 2.9464 | 101.8107 | 2.9936 | 101.7672 | 3.0052 | 101.7577 | 2.9958 | 101.8152 |
| 2.9712 | 101.8168 | 2.9962 | 101.7397 | 3.0041 | 101.7524 | 3.0063 | 101.7951 |
| 2.9665 | 101.811 | 2.9978 | 101.7604 | 3.0622 | 101.8057 | 2.9985 | 101.8153 |
| 2.9674 | 101.7857 | 2.9868 | 101.7545 | 3.0384 | 101.8168 | 2.9955 | 101.7754 |
| 2.9464 | 101.8093 | 3.0081 | 101.7446 | 3.0215 | 101.7788 | 3.0337 | 101.7724 |
| 2.9561 | 101.8008 | 2.9867 | 101.757 | 3.0537 | 101.8067 | 3.0588 | 101.7843 |
| 2.9684 | 101.8008 | 2.9963 | 101.7413 | 3.0282 | 101.7792 | 3.062 | 101.7973 |
| 2.9711 | 101.7892 | 3.0065 | 101.7605 | 3.03 | 101.8166 | 3.0024 | 101.795 |
| 2.9594 | 101.8143 | 2.9953 | 101.758 | 3.0404 | 101.8077 | 3.0241 | 101.8124 |
| 2.9391 | 101.7967 | 3.001 | 101.739 | 3.0145 | 101.7899 | 3.0152 | 101.7954 |
| 2.9588 | 101.7939 | 3.0063 | 101.739 | 3.0039 | 101.8043 | 3.0275 | 101.7903 |
| 2.9332 | 101.7973 | 3.008 | 101.7423 | 3.0321 | 101.7934 | 3.006 | 101.7955 |
| 2.9479 | 101.7985 | 3.0092 | 101.7377 | 3.056 | 101.808 | 2.9897 | 101.8035 |
| 2.9593 | 101.8047 | 2.99 | 101.7522 | 3.0178 | 101.7868 | 3.0202 | 101.7708 |
| 2.9711 | 101.8052 | 2.9917 | 101.7661 | 2.9933 | 101.7734 | 2.9984 | 101.8076 |
| 2.9657 | 101.7992 | 3.0083 | 101.7586 | 3.0208 | 101.7978 | 2.9862 | 101.7767 |
| 2.9513 | 101.7993 | 3.0004 | 101.7552 | 3.0089 | 101.8128 | 3.0632 | 101.7925 |
| 2.9633 | 101.8036 | 2.9981 | 101.7672 | 3.0173 | 101.7791 | 3.0197 | 101.7821 |
| **Latitude** | **Longitude** | **Latitude** | **Longitude** | **Latitude** | **Longitude** | **Latitude** | **Longitude** |
| 3.0638 | 101.7873 | 3.052 | 101.7714 | 3.0323 | 101.776 | 3.0071 | 101.7915 |
| 3.0473 | 101.8011 | 3.0444 | 101.7798 | 3.041 | 101.7809 | 3.046 | 101.7991 |
| 2.9846 | 101.778 | 2.9986 | 101.7914 | 3.0437 | 101.7711 | 3.0037 | 101.8138 |
| 3.0404 | 101.7831 | 3.0139 | 101.776 | 3.038 | 101.7985 | 3.0635 | 101.8092 |
| 3.0426 | 101.7793 | 2.9997 | 101.7704 | 3.0443 | 101.7752 | 3.0355 | 101.8121 |
| 3.0375 | 101.7792 | 2.9841 | 101.8042 | 3.015 | 101.7892 | 3.0338 | 101.7974 |
| 3.0298 | 101.7854 | 3.0103 | 101.7866 | 3.0323 | 101.8116 | 2.9983 | 101.7974 |
| 3.0021 | 101.8114 | 3.0421 | 101.8067 | 2.9936 | 101.7958 | 2.9915 | 101.8102 |
| 3.0481 | 101.7921 | 3.0359 | 101.7905 | 2.9888 | 101.7873 | 3.0052 | 101.7716 |
| 3.0029 | 101.789 | 3.0291 | 101.7905 | 3.0653 | 101.7798 | 3.0553 | 101.8116 |
| 3.0148 | 101.7784 | 3.0204 | 101.7723 | 3.0076 | 101.7907 | 3.0596 | 101.7892 |
| 3.0579 | 101.8155 | 3.0079 | 101.7723 | 3.0334 | 101.8149 | 3.0421 | 101.7717 |
| 3.0551 | 101.7892 | 3.0256 | 101.7743 | 3.0639 | 101.7758 | 3.0442 | 101.8051 |
| 3.0174 | 101.8097 | 3.0472 | 101.7979 | 2.9994 | 101.7921 | 3.0031 | 101.7773 |
| 3.0104 | 101.7989 | 3.0473 | 101.7813 | 3 | 101.8103 | 3.0318 | 101.7768 |
| 3.0345 | 101.7877 | 3.0318 | 101.8095 | 3.0124 | 101.772 | 3.0513 | 101.7985 |
| 3.0595 | 101.8112 | 3.0461 | 101.8103 | 3.0614 | 101.8025 | 3.0175 | 101.782 |
| 3.0595 | 101.8069 | 3.0376 | 101.8153 | 3.0164 | 101.816 | 3.066 | 101.7852 |
| 3.0331 | 101.7919 | 2.9942 | 101.793 | 3.0067 | 101.7833 | 2.9915 | 101.7889 |
| 3.0116 | 101.8083 | 3.0259 | 101.7804 | 2.9966 | 101.7763 | 3.0106 | 101.7891 |
| 3.0548 | 101.8122 | 3.0128 | 101.7806 | 3.017 | 101.8022 | 3.0264 | 101.7882 |
| 3.0207 | 101.7902 | 2.9916 | 101.7952 | 3.0151 | 101.8127 | 2.989 | 101.7987 |
| 3.0591 | 101.7857 | 2.9963 | 101.8058 | 2.9949 | 101.7987 | 3.0442 | 101.7778 |
| 2.9868 | 101.798 | 3.0004 | 101.7863 | 3.0201 | 101.8123 | 3.0302 | 101.7788 |
| 3.0282 | 101.8124 | 3.0398 | 101.7917 | 2.9916 | 101.7791 | 3.0279 | 101.7744 |
| 3.0435 | 101.803 | 3.0198 | 101.8 | 3.035 | 101.8055 | 3.0529 | 101.7852 |
| 2.9989 | 101.7877 | 3.0416 | 101.8131 | 2.9849 | 101.7863 | 3.0553 | 101.8062 |
| 3.0119 | 101.8045 | 3.0053 | 101.7776 | 3.0316 | 101.7897 | 3.0495 | 101.781 |
| 2.9996 | 101.8148 | 2.9848 | 101.8036 | 3.0495 | 101.7773 | 3.0104 | 101.8048 |
| 3.0107 | 101.7955 | 3.0282 | 101.7972 | 3.0035 | 101.8085 | 3.0215 | 101.8026 |
| 3.0175 | 101.7954 | 3.0072 | 101.7904 | 3.0212 | 101.7994 | 3.0464 | 101.8087 |
| 3.0295 | 101.7846 | 3.0625 | 101.8116 | 3.0313 | 101.8047 | 2.9931 | 101.8089 |
| 2.988 | 101.7733 | 3.0592 | 101.7885 | 2.9891 | 101.8078 | 2.9931 | 101.7838 |
| 3.0299 | 101.7786 | 3.0166 | 101.7784 | 3.0252 | 101.7732 | 3.0064 | 101.7845 |
| 3.0068 | 101.7744 | 2.9861 | 101.7998 | 3.0373 | 101.8147 | 3.0275 | 101.7946 |
| 3.004 | 101.7918 | 3.0397 | 101.7993 | 3.0024 | 101.7934 | 3.0647 | 101.7853 |
| 3.0042 | 101.7704 | 3.0535 | 101.7854 | 3.0535 | 101.8055 | 3.043 | 101.8091 |
| 2.9968 | 101.813 | 3.0646 | 101.8077 | 3.0646 | 101.8049 | 3.0099 | 101.8081 |
| 3.0634 | 101.8002 | 2.9887 | 101.817 | 3.0542 | 101.8091 | 3.0082 | 101.7962 |
| 3.0617 | 101.7701 | 3.0214 | 101.8161 | 3.026 | 101.7774 | 3.0546 | 101.7824 |
| **Latitude** | **Longitude** | **Latitude** | **Longitude** | **Latitude** | **Longitude** | **Latitude** | **Longitude** |
| 3.0597 | 101.802 | 3.0327 | 101.7895 | 3.0536 | 101.7953 | 3.0325 | 101.781 |
| 3.0371 | 101.781 | 3.0184 | 101.8044 | 3.0325 | 101.8166 | 3.0077 | 101.7948 |
| 3.0052 | 101.7915 | 3.0097 | 101.8067 | 3.0627 | 101.8055 | 3.0527 | 101.7724 |
| 2.9914 | 101.7881 | 3.0059 | 101.7873 | 2.9891 | 101.8161 | 2.9999 | 101.8056 |
| 3.0167 | 101.8103 | 3.0104 | 101.77 | 2.9981 | 101.7853 | 3.0207 | 101.7983 |
| 3.0526 | 101.8164 | 2.9951 | 101.7954 | 2.9963 | 101.7745 | 3.0298 | 101.7955 |
| 3.0402 | 101.8137 | 3.0397 | 101.7798 | 3.0235 | 101.8051 | 2.9867 | 101.7859 |
| 3.0012 | 101.7892 | 3.0314 | 101.7803 | 3.0594 | 101.8052 | 2.9885 | 101.8091 |
| 3.0508 | 101.796 | 3.0495 | 101.7868 | 3.0431 | 101.7993 | 3.038 | 101.7759 |
| 3.0215 | 101.815 | 3.0142 | 101.7957 | 3.0563 | 101.8074 | 3.0649 | 101.8087 |
| 3.0158 | 101.812 | 3.0282 | 101.7863 | 3.0113 | 101.8051 | 2.9903 | 101.7712 |

**S1 Table B.** Rapid charging stations (RCSs) location addresses

| **Latitude** | **Longitude** |
| --- | --- |
| 2.946 | 101.691 |
| 2.977 | 101.696 |
| 2.973 | 101.721 |
| 3.003 | 101.726 |
| 3.013 | 101.7 |
| 3.027 | 101.725 |
| 3.048 | 101.706 |
| 3.056 | 101.732 |
| 3.041 | 101.746 |
| 2.981 | 101.742 |
| 2.955 | 101.751 |
| 2.928 | 101.767 |
| 2.951 | 101.788 |
| 2.983 | 101.763 |
| 3.019 | 101.772 |
| 3.037 | 101.772 |
| 3.061 | 101.775 |
| 3.036 | 101.8 |
| 3.013 | 101.799 |
| 2.977 | 101.796 |
